# Identification and Validation of Stable Loci Underlying Productivity-Related Traits in Common Wheat

**DOI:** 10.3390/ijms27115130

**Published:** 2026-06-05

**Authors:** Antonina A. Kiseleva, Irina N. Leonova, Mikhail A. Nesterov, Vyacheslav V. Piskarev, Elena A. Salina

**Affiliations:** 1 The Federal Research Center, Institute of Cytology and Genetics, Siberian Branch of the Russian Academy of Sciences, Novosibirsk 630090, Russia; leonova@bionet.nsc.ru (I.N.L.); mikkanestor@bionet.nsc.ru (M.A.N.); salina@bionet.nsc.ru (E.A.S.); 2 Kurchatov Genomics Center, Institute of Cytology and Genetics, Siberian Branch of the Russian Academy of Sciences, Novosibirsk 630090, Russia; 3 Siberian Research Institute of Crop Production and Breeding, Siberian Branch of Russian Academy of Sciences, Novosibirsk 630501, Russia; piskarev@bionet.nsc.ru

**Keywords:** common wheat, grain weight, grain number, thousand-grain weight, QTL, GWAS, KASP

## Abstract

The genetic architecture of wheat yield-related traits is complex due to their polygenic control, relatively low heritability, and strong genotype–environment interactions. Commonly used characteristics of wheat productivity include thousand-grain weight (TGW), grain weight per ear (GWE), and grain number per ear (GNE). To identify stable loci associated with productivity-related traits in common wheat, we performed QTL analysis using two mapping populations derived from crosses between contrasting cultivars. The populations were phenotyped for GNE, GWE, and TGW over two years. In addition, GWAS was conducted using a cultivar panel phenotyped for yield and GWE over ten years, and for GNE, GWE, and TGW over two years. The most reproducible loci were located on chromosomes 2D, 4A, 5A, 5B, 6A, 6B, and 7A. From these regions, 16 SNPs were selected for KASP marker development. Validation in an independent panel of 296 spring common wheat varieties phenotyped over three years identified three most informative markers: wsnp_Ex_c16175_24619793 (4A), wsnp_Ex_c2171_4072995 (5A), and BS00034554_51 (6B), all consistently associated with TGW and additionally associated with GWE, GNE, or yield in individual years. These markers may be useful for marker-assisted selection of wheat productivity-related traits.

## 1. Introduction

Wheat (*Triticum aestivum* L.) is one of the most important cereal crops worldwide, providing approximately 20% of the calories and protein consumed by the global population [1]. To meet the current demands of the growing global population, it is necessary to develop cultivars that exhibit high adaptability and are capable of producing optimal yields under adverse environmental conditions.

Grain yield is a complex quantitative trait controlled by multiple genes and strongly influenced by environmental factors. Many agronomic traits, such as plant height, spike length, number of productive tillers, number of spikelets per spike, grain number and grain weight per spike, thousand kernel weight, and resistance to pathogens, contribute to yield improvement; however, strong and stable correlations between yield and these traits are not always observed. For instance, genotypes with reduced plant height are generally more resistant to lodging, yet the relationship between stem length and yield is not always straightforward, as other stem characteristics (e.g., the diameter of upper and lower internodes and stem wall thickness) may also contribute to lodging resistance [2]. Relatively low correlations have also been reported between yield and traits such as the number of spikelets per spike and the number of grains per spikelet [3]. In addition, spike number per unit area and the number of productive tillers are also considered important yield-related parameters although these traits contribute more substantially to yield formation in winter wheat compared to spring wheat [4,5].

The genetic dissection of yield-related traits has long been challenging due to their polygenic nature, relatively low heritability, and strong genotype–environment (G × E) interactions [6]. However, recent advances in high-throughput genotyping technologies, improved statistical approaches, and large-scale multi-environment trials have enabled researchers to identify and validate reproducible loci across diverse genetic backgrounds and environmental conditions [7].

Many genes associated with wheat productivity traits influence yield-related traits indirectly through developmental processes such as plant height and flowering time.

A well-known example is the *Reduced height-1* (*Rht-1*) gene family, which is primarily controls plant height but also affects grain yield and yield components through changes in spikelet fertility and plant architecture [8,9,10,11]. Another important regulator is *Photoperiod-1* (*Ppd-1*), a key determinant of heading time in wheat. The photoperiod-insensitive allele *Ppd-D1a* accelerates heading but is also associated with changes in yield-related traits, including TGW, GNE, and GWE [12,13]. These examples illustrate the complex and pleiotropic genetic control of wheat productivity-related traits. In contrast, the allele *Vrn-B3a*, another gene variant associated with earlier heading, does not exhibit such a pronounced effect on yield-related traits [12]. However, its homoeolog located on chromosome 7D has been associated with traits such as grain yield, spikelet number, and thousand grain weight [14].

However, a number of genes have been identified that are primarily associated with yield-related traits. These include *TaGW2* [15,16], *TaGS1* [17], *TaGNI* [18,19], *TaCKX6-D1* [20], *TaGS5* [21,22], *TaDA1* [23,24], *TaTGW-7A* [25], *WAPO1* [26,27], *TaODORANT1* [28], *TaWUS-like-5D* [29], and *TaRGN-D1* [30]. Notably, many of these genes are simultaneously associated with several yield-related traits, such as grain size, grain weight, and thousand grain weight. Nevertheless, even well-characterized genes may exhibit different effects depending on environmental conditions.

In addition to individual genes, a large number of quantitative trait loci (QTL) and marker-trait associations (MTA) affecting yield components have been identified using quantitative genetic approaches [7]. These loci are distributed across all 21 chromosomes of bread wheat [31]. Although several so-called “QTL-rich clusters” have been identified, the reproducibility of these loci still strongly depends on cultivation conditions, weather variability, and the genetic background of the studied germplasm. The number of studies reporting novel loci and molecular markers associated with yield traits continues to increase each year.

Improving grain yield component traits remains a primary objective of wheat breeding programs worldwide. The identification of stable and reproducible quantitative trait loci associated with yield traits is therefore essential for understanding the genetic architecture of productivity-related traits and for facilitating the implementation of marker-assisted selection (MAS) and genomic selection strategies in breeding programs. In studies investigating wheat yield and its components, traits such as thousand-grain weight (TGW, also referred to as thousand-kernel weight, TKW), grain weight per ear (GWE, grain weight per spike, GWS), and grain number per ear (GNE, grain number per spike, GNS) are commonly analyzed [32]. TGW is widely considered an important yield-related trait and a valuable target for wheat breeding [4]. However, its contribution to grain yield may depend on environmental conditions, genetic background, and compensatory relationships among yield components.

Two major approaches are commonly used for the identification of loci associated with agronomic traits in wheat: genome-wide association studies (GWAS) and QTL mapping [33]. GWAS exploits linkage disequilibrium in diverse germplasm panels and provides high mapping resolution, allowing rapid detection of marker-trait associations [34]. However, its efficiency depends on population structure, trait heritability, and marker density. In contrast, QTL mapping is based on biparental populations derived from contrasting parental lines and enables reliable detection of loci segregating within a defined genetic background, although with lower mapping resolution [35]. These approaches are therefore considered complementary, and their combined application improves the reliability of identified loci and facilitates marker validation for marker-assisted selection [36,37,38].

Studies on the productivity-related traits of Russian spring wheat cultivars and the analysis of the genetic architecture of yield remain limited and are mainly focused on identifying correlations between yield and other agronomically important traits [39]. The aim of the present study was to identify stable and reproducible loci associated with productivity-related traits in spring bread wheat using a diverse panel of plant material developed or cultivated in Russia.

## 2. Results

### 2.1. Phenotypic Variation in the Studied Collections

To identify loci associated with productivity-related traits in common wheat, the following plant material was used: (1) a mapping population derived from the cross Obskaya 2 × Novosibirskaya 15; (2) a mapping population derived from the cross Obskaya 2 × Tulun 15; and (3) a collection of 92 spring common wheat varieties (Panel 1).

Phenotypic data for grain number per ear (GNE), grain weight per ear (GWE), and thousand grain weight (TGW) were obtained for all three panels during 2017–2018 in Novosibirsk region. In addition, long-term phenotypic data for GWE and yield (centner/ha) collected over 10 years (2005–2014) were available for Panel 1. Based on these data, for the mapping populations, genetic linkage maps were constructed, and QTL analysis for the studied traits was performed. A genome-wide association study (GWAS) was performed for Panel 1.

#### 2.1.1. Phenotypic Variation in Obskaya 2 × Novosibirskaya 15

Descriptive statistics for GNE, GWE, and TGW in the F_2:3_ population derived from the cross Obskaya 2 × Novosibirskaya 15 (Ob2 × N15) revealed substantial phenotypic variation across both years of evaluation (Table S1). Mean GNE increased from 25.23 in 2017 to 28.60 in 2018, while mean GWE increased from 0.78 to 1.14 g. TGW also showed a marked increase between years, from 30.70 g in 2017 to 40.05 g in 2018. The coefficients of variation ranged from 6.70% for TGW in 2018 to 15.92% for GWE in 2017, indicating low-to-moderate phenotypic variability, with the highest variation observed for GWE.

The Shapiro–Wilk test showed that all traits followed a normal distribution in both years (Table S1). Correlation analysis revealed a strong positive association between GNE and GWE in both 2017 (r = 0.82, *p* < 0.001) and 2018 (r = 0.85, *p* < 0.001), indicating that grain number per spike was closely related to spike grain weight (Figure S1a). GWE was also significantly and positively correlated with TGW in 2017 (r = 0.62, *p* < 0.001) and 2018 (r = 0.46, *p* < 0.001). In contrast, correlations between GNE and TGW were weak and non-significant in both years. Significant correlations across years were observed for GNE (r = 0.58, *p* < 0.001), GWE (r = 0.47, *p* < 0.001), and TGW (r = 0.55, *p* < 0.001), suggesting moderate stability of trait expression across years.

#### 2.1.2. Phenotypic Variation in Obskaya 2 × Tulun 15

GNE, GWE, and TGW in the F_2:3_ population derived from the cross Obskaya 2 × Tulun 15 considerably varied across the two years of evaluation (Table S1). Mean GNE increased from 22.47 in 2017 to 26.06 in 2018, while mean GWE increased from 0.75 to 1.16 g. TGW also showed a pronounced increase, from 33.12 g in 2017 to 44.72 g in 2018. Most traits followed a normal distribution (*p* > 0.05), although GNE in 2017 deviated from normality (Table S1).

Correlation analysis revealed a strong positive relationship between GNE and GWE in both years, with correlation coefficients of r = 0.85 in 2017 and r = 0.81 in 2018 (*p* < 0.001), suggesting that grain number per spike was the major contributor to spike productivity (Figure S1b). GWE was also positively correlated with TGW in 2017 (r = 0.51, *p* < 0.001) and 2018 (r = 0.50, *p* < 0.001). In contrast, correlations between GNE and TGW were weak and non-significant in both years. Significant positive correlations between years were detected for GNE (r = 0.61, *p* < 0.001), GWE (r = 0.55, *p* < 0.001), and TGW (r = 0.49, *p* < 0.001), indicating moderate stability of trait expression across years.

#### 2.1.3. Yield and GWE in Panel 1 in 2005–2014 Years

GWE and Yield varied substantially across years through the 10-year evaluation period, reflecting the strong influence of environmental conditions on productivity-related traits (Table S2). Mean GWE values ranged from 1.06 g in 2005–2006 to 1.38 g in 2014, whereas Yield varied more markedly, from 11.1 g in 2012 to 40.8 g in 2013. The coefficient of variation for GWE ranged from 13.9% to 21.9%, indicating relatively stable expression of this trait, while Yield exhibited substantially broader variability (14.4–48.1%), suggesting stronger environmental sensitivity and genotype-by-environment interactions. The highest Yield values were recorded in 2013–2014, whereas lower productivity was observed in 2005, 2007, and especially 2012.

Normality testing demonstrated that distribution patterns differed among years and between traits (Table S2, Figure S2a). GWE showed distributions close to normality in most years, although deviations were detected in 2005, 2009, 2012, and 2013. Yield exhibited less stable distribution patterns, with normality confirmed only for 2006, 2010, and 2011, while the remaining years showed deviations from normal distribution (*p* < 0.05).

Correlation analysis revealed predominantly positive relationships across yeas for both traits, indicating partial stability of genotype performance in contrasting environments (Figure S3a). Yield displayed moderate correlations between years. GWE correlations were generally weaker but remained mostly positive. Cross-trait correlations between Yield and GWE were mostly low to moderate, although several significant positive relationships were observed in favorable years. Collectively, these results indicate moderate phenotypic consistency across years together with a substantial contribution of environmental factors to the expression of productivity-related traits.

Broad-sense heritability was 0.47 for GWE and 0.39 for Yield, indicating a moderate contribution of the genetic component to the formation of both traits.

#### 2.1.4. GNE, GWE and TGW Variation in Panel 1 in 2017–2018 Years

Descriptive statistics demonstrated moderate phenotypic variation for all three productivity-related traits across the two years of evaluation, reflecting both genetic diversity and environmental effects on yield component formation (Table S2). GNE showed relatively stable performance, with mean values increasing slightly from 29.3 in 2017 to 30.8 in 2018. The coefficient of variation remained low and nearly identical between years (11.5%), indicating comparatively stable expression and limited environmental sensitivity of this trait. GWE exhibited more pronounced interannual variation, with mean values increasing from 0.83 g in 2017 to 1.20 g in 2018. TGW also differed substantially between years, with mean values of 27.8 g in 2017 and 39.0 g in 2018. Despite this shift, TGW demonstrated the broadest phenotypic range among the evaluated traits. Normality testing showed that most trait distributions did not deviate from normality (Table S2, Figure S2b).

Correlation analysis revealed predominantly positive and statistically significant relationships among productivity-related traits, indicating stable trait interactions across environments (Figure S3b). GNE demonstrated moderate interannual stability between 2017 and 2018 (r = 0.471, *p* < 0.001). Strong positive correlations were observed between GNE and GWE within both years, including GNE_2017–GWE_2017 (r = 0.734, *p* < 0.001) and GNE_2018–GWE_2018 (r = 0.793, *p* < 0.001), suggesting that grain number per ear was a major determinant of ear productivity. GWE also showed significant interannual correlation (r = 0.606, *p* < 0.001), reflecting moderate stability of this trait.

TGW demonstrated strong consistency across years (r = 0.736, *p* < 0.001) and moderate-to-strong positive correlations with GWE (r = 0.509–0.751, *p* < 0.001), indicating a substantial contribution of grain filling efficiency to grain weight per ear. In contrast, correlations between TGW and GNE were weak and non-significant (r = 0.011–0.132), suggesting relative independence between grain number formation and grain size. Collectively, these results indicate that GWE is jointly determined by both grain number and grain weight components, whereas GNE and TGW contribute to productivity through largely independent mechanisms.

Broad-sense heritability was 0.58 for GWE, 0.47 for GNE and 0.73 for TGW, indicating a moderate genetic contribution to the variation in GWE and GNE and a high heritability level for TGW.

### 2.2. Genetic Mapping and QTL Analysis

For the Ob2 × N15 (Obskaya 2 × Novosibirskaya 15) population, 2350 polymorphic markers were initially identified, of which 1355 were successfully mapped. Among these, 398 were classified as skeletal markers, each representing a set of co-segregating loci. Overall, 27 linkage groups were obtained. Several chromosomes (1B, 3B, 5A, 7A, and 7B) were split into more than one linkage group (Figure S4).

In the Ob2 × T15 (Obskaya 2 × Tulun 15) population, 3323 polymorphic markers were detected, and 2629 of them were successfully mapped, including 534 skeletal markers. This resulted in 26 linkage groups, with chromosomes 3B, 3D, 5D, and 7D each represented by multiple linkage groups (Figure S5).

In both populations, the D genome was noticeably underrepresented compared to the A and B genomes. In the Ob2 × N15 population, chromosomes of the A and B genomes contained on average 22–24 skeletal markers (corresponding to 63–97 total markers), whereas the D genome was represented by only about 9 skeletal markers per chromosome (33 markers in total). A comparable trend was observed in the Ob2 × T15 population: chromosomes of the A and B genomes harbored on average 32 skeletal markers (126–208 total markers), while the D genome included approximately 11 skeletal markers per chromosome (40 markers in total). This imbalance likely reflects limitations of the genotyping platform, as markers targeting the D genome are generally less abundant than those designed for the A and B genomes.

#### 2.2.1. QTL Mapping of Obskaya 2 × Novosibirskaya 15

**Figure 1:**
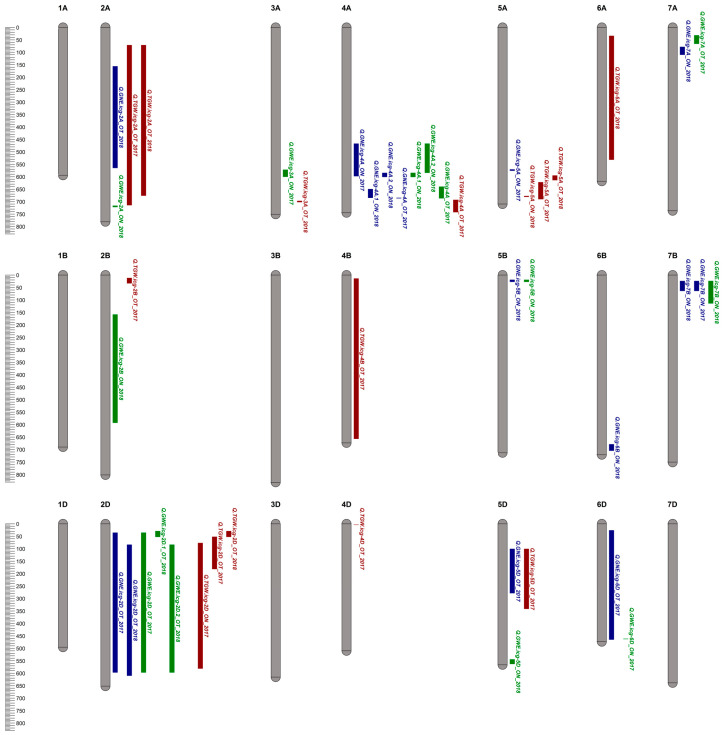
Localization of QTLs identified in the biparental populations Ob2 × N15 (ON) and Ob2 × T15 (OT) on the physical map (RefSeq v1.1). Blue indicates loci associated with GNE, green with GWE, and red with TGW.

QTL mapping in the Ob2 × N15 population identified several loci associated with grain-related traits in both growing seasons (2017 and 2018) (Table 1, Figure 1, Table S3).

In 2017, six significant QTLs were detected. Three of them were associated with grain number per ear (GNE) and were located on chromosomes 4A, 5A, and 7BS. For grain weight per ear (GWE), two loci were identified on chromosomes 6D and 3A. In addition, a single QTL affecting thousand grain weight (TGW) was mapped to chromosome 2D.

In contrast, the 2018 season showed a higher number of detected loci, with fourteen QTLs identified overall. For GNE, seven loci were found, including two on chromosome 4A and one each on 7A, 5B, 6B, and 7BS. For GWE, six QTLs were mapped to chromosomes 2A, 4A (two loci), 2B, 5B, 7B, and 5D. One locus associated with TGW was detected on chromosome 5A.

Across years, several genomic regions showed consistent or overlapping effects. A stable QTL for GNE was identified on chromosome 7BS in both 2017 and 2018, localized to the same marker interval (Ex_c101666_634-BS00010616_51), indicating a robust and repeatable genetic effect. In addition, chromosome 4A harbored multiple QTLs for both GNE and GWE in 2018, and also contained a GNE locus in 2017, suggesting that this chromosome represents a hotspot for loci controlling grain productivity traits in this population.

Furthermore, co-localization of QTLs for different traits was observed. In particular, overlapping regions on chromosome 4A were associated with both GNE and GWE in 2018, implying either pleiotropic effects or tight linkage of genes controlling these traits. Similarly, the region on chromosome 5B was associated with both GNE and GWE in 2018, further supporting the presence of shared genetic control.

Overall, the results demonstrate both year-specific and stable QTLs, with several chromosomal regions (notably 4A, 5B, and 7BS) contributing to multiple yield-related traits.

#### 2.2.2. QTL Analysis of Obskaya 2 × Tulun 15

QTL analysis of the Ob2 × T15 population also revealed a substantial number of significant loci for grain-related traits across two growing seasons (2017 and 2018) (Table 2, Figure 1, Table S4).

In 2017, fourteen QTLs associated with grain-related traits (GNE, GWE, and TGW) were detected. For GNE, five loci were mapped to chromosomes 4A, 2D, 5D, and 6D; three of these showed positive effects contributed by the Obskaya 2 allele, whereas the remaining two were associated with the Tulun 15 allele. For GWE, three loci were identified on chromosomes 4A, 7A, and 2D. TGW was controlled by eight QTLs located on chromosomes 2A, 4A, 5A, 2B, 4B, 2D, 4D, and 5D. Most of these loci were associated with positive effects from Obskaya 2, except for the locus on chromosome 2B, where the Tulun 15 allele had a favorable effect.

In 2018, additional loci were detected, resulting in a total of eleven QTLs across the three traits. For GNE, two loci were identified on chromosomes 2A and 2D, with favorable effects contributed by Obskaya 2 and Tulun 15, respectively. For GWE, three QTLs were mapped to chromosome 2D, all associated with positive effects from Obskaya 2. In the case of TGW, five loci were detected on chromosomes 2A, 5A, 6A, 2D, and 3A, again predominantly reflecting contributions from the Obskaya 2 allele.

Several genomic regions showed consistent effects across years. In particular, chromosome 2D harbored QTLs associated with both GNE and GWE in 2017 and 2018, indicating a possible role in the coordinated genetic control of these traits. Likewise, chromosome 2A exhibited stable QTLs for TGW in both years and additionally carried a GNE-associated locus in 2018.

Co-localization of QTLs for different traits was evident in multiple regions. This was especially pronounced on chromosome 2D, where loci affecting both GNE and GWE were detected in both seasons. Chromosome 4A also contained several QTLs linked to GNE and TGW across years, further supporting its importance in regulating grain-related traits in this population.

Overall, the results indicate the presence of both environment-specific and stable QTLs, with chromosomes 2D, 4A, and 5A emerging as key regions potentially involved in the genetic control of yield components in wheat.

A comparison of QTL profiles between the two populations (Ob2 × N15 and Ob2 × T15) revealed shared and population-specific loci underlying grain-related traits. In general, the Ob2 × T15 population showed a greater number of detected QTLs, particularly for TGW, whereas Ob2 × N15 was characterized by fewer loci, some of which were more consistently detected across years, especially for GNE.

Several chromosomal regions were common to both populations. For instance, chromosome 4A carried multiple QTLs in each case (Figure 1). In Ob2 × N15, loci associated with GNE and GWE were mapped to this chromosome, while in Ob2 × T15, QTLs affecting all three traits (GNE, GWE, and TGW) were identified. This pattern suggests that chromosome 4A may play a central role in the genetic control of grain productivity traits across different genetic backgrounds.

Chromosome 2D also showed repeated involvement. In Ob2 × N15, a TGW-related locus was detected in 2017, whereas in Ob2 × T15, this chromosome harbored multiple QTLs associated with GNE, GWE, and TGW in both years. The recurrence of signals on chromosome 2D in both populations points to a potentially stable genetic factor influencing yield components.

A similar trend was observed for chromosome 5A, which was linked to TGW in both populations (in 2018 for Ob2 × N15 and in both years for Ob2 × T15), indicating partial conservation of the genetic basis of grain weight. Chromosome 5B also appeared noteworthy: in Ob2 × N15, overlapping QTLs for GNE and GWE were detected in 2018, whereas in Ob2 × T15, loci associated with TGW were identified in 2017, suggesting possible pleiotropic or closely linked effects.

At the same time, some loci appeared to be population-specific. For example, a stable QTL for GNE on chromosome 7BS was detected only in Ob2 × N15 across both years and was not observed in Ob2 × T15. In contrast, chromosomes such as 2A, 4B, 4D, and 6A were more prominently associated with trait variation in the Ob2 × T15 population, particularly in relation to TGW.

### 2.3. GWAS Based on a Spring Common Wheat Panel 1 Across 10 Years of Phenotyping

Yield (centners per hectare) and grain weight per ear were assessed for ten years (2005–2014). A GWAS identified 97 SNPs significantly associated with yield at a threshold of 0.001. These associations were distributed across nearly all chromosomes, with the exception of 3D, 4B, 4D, and 7D, and were most densely concentrated on chromosomes 1B, 6A, 2D, and 5A (Table S5, Figures S6 and S8).

Analysis of linkage disequilibrium indicated that while some marker-trait associations (MTAs) were represented by individual SNPs, most significant markers related to TGW formed clusters of 2–6 SNPs, corresponding to distinct loci (Figure S7, Table S6).

Several genomic regions exhibited reproducible associations across years. For example, a region on chromosome 1B (15–16 Mb) was repeatedly associated with yield in 2005 and 2007, supported by multiple closely linked markers (e.g., BS00089524_51, BS00011695_51), suggesting a stable QTL. Another region on the same chromosome (687–688 Mb) showed consistent associations in 2009 and 2011, further indicating its robustness across environments.

Chromosome 2D also harbored an important region. In particular, in 2011, a cluster of tightly linked markers spanning 108–159 Mb was identified, explaining up to approximately 19–20% of the phenotypic variance. Associations on this chromosome were also observed in other years, supporting its role in yield formation.

A prominent hotspot was detected on chromosome 6A, especially in 2005, where a dense group of highly significant markers was localized in the 611–613 Mb interval. This region showed the strongest signals in the dataset (*p*-values down to 3.19 × 10^−6^) and accounted for up to 25% of the phenotypic variance, indicating the presence of a major QTL.

Chromosome 5A also contributed several associations across different years (2006, 2011, and 2012), suggesting moderate stability of this region. Likewise, chromosome 4A contained two distinct loci (at ~10 Mb and 584–606 Mb). The locus near 10 Mb was detected in both 2012 and 2014, indicating a consistent effect.

Overall, the GWAS results indicate that yield is governed by multiple loci with varying effect sizes, including several relatively stable genomic regions (notably on chromosomes 1B, 4A, and 6A), as well as numerous environment-dependent associations, reflecting pronounced genotype × environment interactions.

The analysis of GWE identified 57 SNPs significantly associated with this trait at a threshold of 0.001, distributed across chromosomes 1A, 1B, 1D, 2A, 2B, 2D, 3A, 3B, 4A, 4B, 4D, 5A, 5B, 6A, 6D, 7A, 7B, and 7D (Table S7). Linkage disequilibrium patterns suggested that many of these SNPs were not independent but rather formed clusters corresponding to loci consisting of 2–8 markers (Figure S9, Table S6). The largest clusters were located on chromosomes 1B (7 SNPs) and 2B (8 SNPs), with pairwise R^2^ values ranging from 0.20 to 0.28.

The highest density of MTAs was observed on chromosomes 1B, 2A, 2B, 4A, and 5A. In particular, chromosome 1B contained several prominent clusters, especially within the 367–424 Mb interval and around 660 Mb, where groups of closely linked markers detected in 2009 formed distinct association peaks with relatively high explained phenotypic variance (up to 30%).

Chromosome 2A also contributed a notable region of associations, most clearly observed in 2009 within a broad interval (688–712 Mb), where several markers showed moderate to strong effects (R^2^ up to 0.28). A comparable pattern was observed on chromosome 2B, which harbored multiple loci, including a dense cluster in the 423–460 Mb region (primarily detected in 2014), suggesting the presence of a relatively stable genomic region affecting GWE.

Several loci exhibited relatively large effects. Markers on chromosomes 1A, 1B, 2A, and 7A reached R^2^ values of 0.28–0.30, indicating major-effect regions. Notably, strong associations were detected on chromosome 1B at 660 Mb and on chromosome 7A at 642 Mb, both corresponding to the highest proportion of explained phenotypic variance.

Evidence for stability across years was also observed. For example, chromosome 5A showed signals in multiple seasons (2007, 2009, and 2013), suggesting a consistent contribution to trait variation. Similarly, chromosome 2B contained loci detected in different years (2010 and 2014), supporting the presence of stable genomic regions influencing GWE.

Co-localization of associations was also evident. On chromosome 4A, a cluster of markers around 713–714 Mb was associated with GNE in 2011, indicating a localized QTL region. Additionally, overlapping signals across different chromosomes (e.g., 1B, 2A, 2B, and 5A) suggest either pleiotropic effects or tightly linked loci contributing to trait variation under different environmental conditions.

Overall, the GWAS results indicate that GNE is controlled by multiple loci of varying effect sizes, including several major-effect regions (notably on chromosomes 1B, 2A, and 7A) and numerous moderate-effect loci, with both stable and environment-specific associations detected across years.

### 2.4. GWAS Based on a Spring Common Wheat Panel 1 for GNE, GWE, and TGW Across Two Years of Evaluation

Genome-wide association analysis for grain number per ear (GNE), grain weight per ear (GWE), and thousand grain weight (TGW) analyzed in 2017 and 2018, was performed (Table S8, Figure S10).

For GNE, significant associations were detected on chromosomes 1A, 1B, 3A, 4B, 5A, 5B, 6A, 6B, 6D, and 7A. Several genomic regions showed consistent effects across years. Notably, stable loci were identified on chromosome 5A (marker Excalibur_c1208_72, detected in both 2017 and 2018) and on chromosomes 6A and 6B, where loci of tightly linked markers were repeatedly detected in both years (Figure S11). In particular, a major region on chromosome 6A (609–615 Mb) contained multiple markers significantly associated with GNE. Additional stable loci were observed on chromosome 6B (e.g., Tdurum_contig62941_85 and Excalibur_c31379_71), suggesting consistent genetic control of GNE in these regions.

For GWE, significant associations were identified on chromosomes 1A, 1B, 1D, 2B, 3A, 3D, 4A, 5A, 5B, 5D, 6A, 6B, and 7D. In contrast to GNE, most associations for GWE were year-specific, although several genomic regions showed co-localization across traits. For example, loci on chromosomes 4A (around 609–624 Mb) and 5B (679 Mb) were associated with both GWE and TGW in 2018. Chromosome 6B harbored a particularly large number of GWE-associated markers in 2017, forming several loci (e.g., 149–236 Mb), indicating an important role of this chromosome in controlling grain weight per ear. Additionally, a strong association was detected on chromosome 3D in 2018 with relatively high R^2^ (~0.20), suggesting a locus with moderate effect.

For TGW, significant associations were detected on chromosomes 1A, 1B, 1D, 2D, 3A, 4A, 4D, 5A, 5B, and 6A. Several loci demonstrated stability across years. In particular, a locus on chromosome 4A (wsnp_Ex_c16175_24619793 at 609 Mb) was associated with TGW in both 2017 and 2018, and also co-localized with GWE in 2018. Another stable and major locus was identified on chromosome 5A (BS00009531_51 at 485 Mb), detected in both years, indicating a strong contribution to grain weight. Chromosome 2D also contained a cluster of closely linked markers (60–61 Mb) associated with TGW in 2018, forming a clear QTL.

### 2.5. Overlapping MTA and QTLs

A comparative analysis integrating QTL mapping results from two biparental populations and GWAS results based on both long-term (10-year) and short-term (2017–2018) phenotyping datasets revealed several overlapping genomic regions controlling yield and its components. Several chromosomes consistently harbored overlapping loci across all datasets, with the most prominent regions located on chromosomes 2D, 4A, 5A, 5B, 6A, and 7A (Table 3).

Chromosome 4A represented a key genomic region, where QTLs for GNE and GWE were identified in both biparental populations, while GWAS detected associations for both yield and GWE over 10 years, as well as for TGW and GWE in the 2017–2018 dataset. The consistent detection of loci within the same genomic interval (580–620 Mb) across traits and approaches indicates a major region controlling wheat productivity-related traits.

Chromosome 5A showed strong and consistent overlap across all analyses. QTLs for TGW were detected in both mapping populations, while GWAS revealed associations for both yield and GWE (10-year dataset) and for all three traits (GNE, GWE, TGW) in the 2017–2018 dataset. This region (~480–680 Mb) appears to be one of the most stable and important loci contributing to yield formation.

A major hotspot was identified on chromosome 6A (600–615 Mb), where GWAS detected a strong yield-associated region across 10 years, and multiple associations for GNE and GWE were identified in the 2017–2018 dataset. In addition, QTLs for GNE and TGW were detected in the Ob2 × T15 population, further supporting the importance of this region.

Chromosome 2D demonstrated strong agreement between QTL and GWAS approaches. QTLs for TGW (in Obskaya 2 × Novosibirskaya 15) and for GNE, GWE, and TGW (in Ob2 × T15) overlapped with GWAS signals for yield and TGW, suggesting a stable genomic region with multi-trait effects.

Chromosome 5B exhibited consistent co-localization of loci for multiple traits across all datasets. Both biparental populations revealed QTLs for GNE and GWE, while GWAS detected associations for yield and GWE (10-year dataset) and for TGW and GWE (2017–2018), particularly in the distal region (680 Mb).

Additional overlapping regions were observed on chromosomes 3A, 6B, and 7A, where loci for different traits were detected across datasets, although with lower consistency compared to the major hotspots.

### 2.6. Phenotyping of the Validation Set of Varieties

The spring common wheat variety panel was grown for three years, from 2022 to 2024, under field conditions in the Novosibirsk region, and phenotyped for key traits related to wheat productivity, including grain weight per ear (GWE), grain number per ear (GNE), thousand-grain weight (TGW), and yield (in c/ha) (Table S9, Figure S12a).

For grain weight per ear (GWE), the mean values in 2022, 2023, and 2024 were 0.87 g, 0.71 g, and 0.98 g, respectively, with the 2024 year showing the highest variation (SD = 0.19) compared to the other years. The distribution for GWE in 2022 and 2024 was slightly skewed, while 2023 exhibited a near-normal distribution.

Grain number per ear (GNE) increased significantly from 2022 (mean = 21.42) to 2024 (mean = 33.69), with the highest variation observed in 2024 (SD = 5.74). The data for GNE followed a normal distribution for 2023 and 2024, with slight skewness observed in 2022.

For thousand-grain weight (TGW), the mean in 2022 was 40.63 g, while 2023 and 2024 showed lower means (35.59 g and 29.18 g, respectively). The variation for TGW in 2024 was higher, as indicated by the increased standard deviation (SD = 3.35). The distribution for TGW in 2023 and 2024 showed slight deviations from normality, particularly for 2024.

Finally, yield in c/ha increased from 392.45 c/ha in 2022 to 451.71 c/ha in 2024, with the largest variation recorded in 2022 (SD = 71.22). Yield values in 2023 and 2024 were more stable, with normal distribution across the years. These results highlight significant year-to-year variability in wheat productivity-related traits, which is expected given the environmental and genetic factors influencing these characteristics.

Correlation analysis based on mean trait values across the three years of evaluation revealed the following relationships among the estimated traits (Figure S12b). GWE showed the strongest positive correlation with GNE (r = 0.780, *p* < 0.001), GWE was also moderately and positively correlated with TGW (r = 0.448, *p* < 0.001) and grain yield (r = 0.538, *p* < 0.001). TGW demonstrated a moderate positive correlation with grain yield (r = 0.445, *p* < 0.001), supporting its relevance as a yield-related trait in the studied spring wheat germplasm. GNE showed only a weak positive correlation with yield (r = 0.261, *p* < 0.001), despite its strong association with GWE. Overall, these results indicate that grain yield in the validation panel was associated with multiple yield components, with GWE showing the strongest relationship with yield, while the contribution of TGW was positive but moderate and partially independent from GNE.

### 2.7. Validation of the Developed KASP Markers in Panel 2 of Spring Common Wheat

From the most stable loci on chromosomes 2D, 4A, 5A, 5B, 6A, 6B, and 7A., 16 SNPs were selected for KASP design. Among the 16 tested KASP markers, 10 were polymorphic and showed precise clustering. Factor analysis was conducted for these markers to identify associations between allele variants of the tested markers and productivity-related traits. Of these 10 markers, three (Excalibur_c31379_71, wsnp_Ex_c1944_3664205, RAC875_c65419_229) were not associated with any of the studied traits. The SNPs GENE-1313_319 (chromosome 2D) and Excalibur_rep_c113131_624 (chromosome 4D) were associated with only one trait (GWE) in one of the study years.

**Figure 2:**
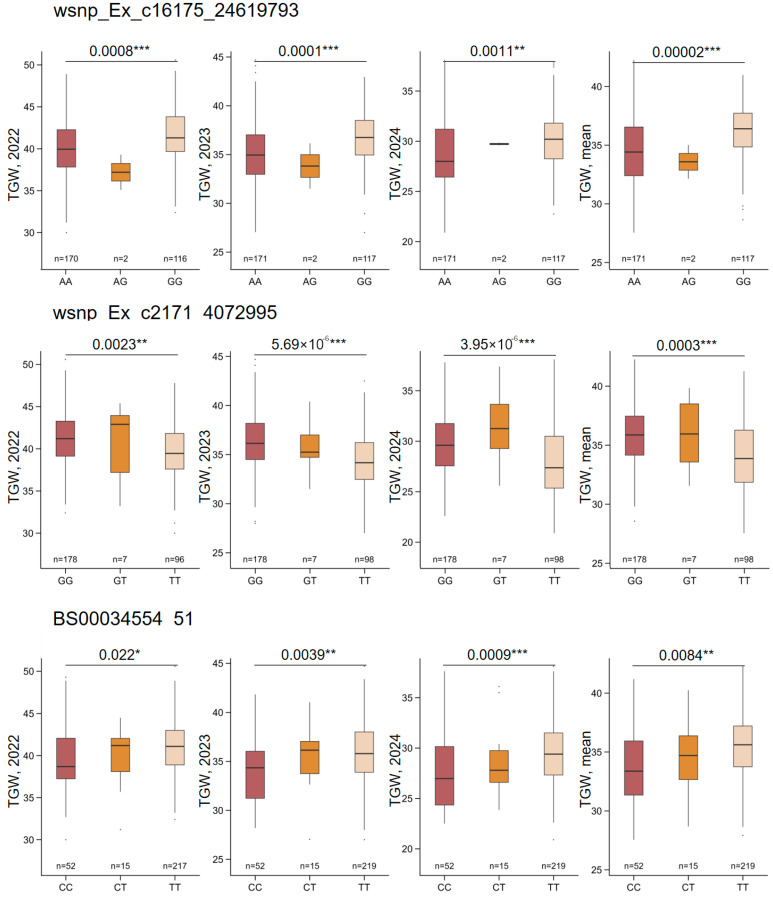
Boxplots showing TGW common wheat varieties (Panel 2) depending on the allele of wsnp_Ex_c16175_24619793, wsnp_Ex_c2171_4072995, and BS00034554_51. The significance level indicated above each box, *—*p*-value < 0.05, **—*p*-value < 0.01, ***—*p*-value < 0.001. The horizontal line inside each box represents the median value. Numbers at the bottom indicate the number of genotypes in each group.

The highest number of significant associations was observed for the KASP markers wsnp_Ex_c16175_24619793 on chromosome 4A, wsnp_Ex_c2171_4072995 on chromosome 5A, and BS00034554_51 on chromosome 6B (Tables S10 and S11, Figure 2). Since TGW was the only trait consistently associated with all three markers across the three years of validation, Figure 2 illustrates the allelic effects of these markers on TGW. Associations with other productivity-related traits were less stable and were detected only in individual years; therefore, they are summarized in Table 4 and presented in detail in Tables S10 and S11.

The marker wsnp_Ex_c16175_24619793 was significantly associated with TGW in all years of the trials at a high level of significance (*p*-value < 0.001 and BH-adjusted *p*-value < 0.05), as well as with GWE in 2022 and 2024. Similarly, wsnp_Ex_c2171_4072995 showed strong and consistent associations with TGW across all years (*p*-value < 0.001 and BH-adjusted *p*-value < 0.05), and additionally with Yield in 2022, GWE in 2024, and GNE in 2023 and 2024.

The marker BS00034554_51 demonstrated less stable associations with TGW, with significance levels ranging from *p*-value < 0.05 to < 0.001 depending on the year, and was also associated with Yield in 2022 and 2023, as well as with GWE in 2024.

Interestingly, BS00023055_51 showed strong associations with Yield in 2022 and 2023 (BH-adjusted *p*-value < 0.05), but did not exhibit any association with TGW.

## 3. Discussion

First part of our study included QTL identification in two biparental populations, Obskaya 2 × Novosibirskaya 15 and Ob2 × T15. In both populations, the variety Obskaya 2 was more often the source of alleles with positive effects on the studied traits. This observation is expected and consistent, as Obskaya 2 is characterized by a high yield potential, whereas the varieties Novosibirskaya 15 and Tulun 15 are distinguished by superior grain quality parameters compared to Obskaya 2. The comparison of loci, significantly associated with yield-related traits in two biparental populations, indicates that while a subset of genomic regions (notably 4A, 2D, and 5A) consistently contributes to grain-related traits across genetic background, a considerable proportion of QTLs is population-specific, reflecting the influence of genetic background and environment on the expression of quantitative traits.

Continuing the work on identification of loci, associated with yield-related traits in plant material developed or cultivated in Russia we performed GWAS using two phenotyping datasets, first describing yield and GWE assessed for long-term period of ten years and second, describing TGW, GNE and GWE assessed for short-term period of two years.

The association analysis revealed a large number of significant marker-trait associations distributed across multiple chromosomes and years, indicating a complex genetic architecture of the trait. Across traits, several genomic regions exhibited co-localization of associations, indicating potential linkage. Notably, chromosomes 4A, 5B, and 6A contained overlapping loci for GNE, GWE, and TGW, suggesting that these regions may harbor key genes controlling multiple yield components. Overall, the GWAS results revealed both stable and environment-specific loci, with several major-effect regions (particularly on chromosomes 4A, 5A, 6A, and 6B) and multiple moderate-effect loci contributing to variation in grain yield components. The results of multi-year trials of a collection of spring common wheat varieties confirm that the expression of yield-related traits and the genetic mechanisms underlying their formation vary considerably from year to year.

### 3.1. Co-Localization with Known Genes Controlling Yield-Related Traits

To determine whether the loci identified in our study overlap with known genes, we compared their positions with those of genes previously reported to be associated with yield-related traits. Both genes directly associated with productivity-related traits and those primarily affecting other traits, such as heading or maturity time and plant or spike architecture, but also shown to influence yield components, were included in the analysis.

#### 3.1.1. Locus on 4A

One of the most striking overlap was observed on chromosome 4A (609–624 Mb), where loci associated with GNE, GWE, and TGW co-localized with *TaCWI-4A* (610 Mb), a gene known to be associated with higher TGW, that aligns with our results [40]. Approximately in the same region on 4A (580–620 Mb), other well-known genes *Rht-A1* (582 Mb) and *TB-A1* (582 Mb) are located. Primarily, these genes are involved in control of plant height and spike architecture, respectively, but they are also known to be associated with yield formation through plant architecture [41,42]. Thus, this locus on the long arm of chromosome 4A harbors multiple well-characterized genes that may collectively control wheat productivity. Therefore, the selection of varieties carrying this entire locus as donors, combining favorable alleles of several productivity-related genes that are naturally stacked within the same chromosomal region, may represent a promising strategy for the development of high-yielding wheat cultivars.

#### 3.1.2. Locus on 5A

On chromosome 5A, several loci overlapped with *TaGL3-5A* (571 Mb) controlling grain size and *TaNAC2-5A* (645 Mb), associated with yield [43,44]. The *TaNAC2-5A* has been reported to be a nitrate-inducible transcription factor, and overexpression of this gene positively influenced root growth, higher concentration of nitrogen in grains, and overall grain yield [44]. So its co-localization with stable associations detected in both GWAS and QTL analyses with TGW and Yield in our study can be considered rather reasonable. Our loci on 5A also overlapped with key developmental genes, including *Vrn-A1* (587 Mb) and *Q* (650 Mb), that were shown to be associated with yield traits [45,46,47,48,49,50]. Therefore, identified region on 5A was consistently associated with TGW and yield across datasets in our study, indicating that variation in flowering time and spike morphology may contribute significantly to grain productivity.

#### 3.1.3. Locus on 2D

Locus on chromosome 2D showed co-localization with *TaSUS2-2D* (119 Mb), a sucrose synthase gene, which haplotypes were shown to segregate cultivars for TGW [51]. In other study, homoeologs of this gene on 2A and 2B chromosome were also associated with TGW [52]. Thus, this gene may represent a likely candidate underlying the effects on TGW, GNE, GWE, and yield within the loci identified in our study. Another overlap on chromosome 2D was associated with *WFZP-2D*, a gene known to control spikelet number per spike, and proposed to improve the grain yield potential of wheat [53].

#### 3.1.4. Additional Loci Overlapping with Individual Yield-Related Genes

A significant overlap was also detected on chromosome 3A, where TGW-associated loci coincided with *TaGS5-3A* (176 Mb), a known regulator of grain size, indicating that this region likely contributes to grain weight variation through modulation of grain development [21,22].

On chromosome 2B, loci associated with GWE overlapped with *Tabas1-B1* (448 Mb) and *TaDA1* (4 Mb), both of which are involved in grain size and weight determination [23,54].

Chromosome 5D showed co-localization with *TaCWI-5D* (557 Mb), another cell wall invertase gene [40]. Interestingly, another gene from this family co-localized with a locus on chromosome 4A, suggesting that carbohydrate metabolism might play an important role in yield variation.

In addition, regions on chromosomes 7A overlapped with *Vrn-A3* (71 Mb). Previously, its homoeolog on chromosome 7D was shown to influence traits such as grain yield, spikelet number, and TGW, suggesting that flowering time pathways also may contribute to variation in yield and grain-related traits [14].

Overall, the observed co-localization of QTLs and MTAs with known genes controlling not only yield characteristics themselves, but also plant development, flowering time, and spike architecture, supports the robustness of the identified loci and indicates that yield variation is largely governed by key regulatory pathways with pleiotropic effects.

### 3.2. Validation of KASP

Based on a comprehensive analysis using multiple approaches (association mapping and QTL analysis), on a panel of domestic cultivars and varieties adapted to Western Siberia, cultivated under the conditions of the Novosibirsk region (Russia), several chromosomal regions were identified that most reliably harbor loci associated with grain number per ear, grain weight per ear, thousand-grain weight, and yield. A comparative analysis integrating the results of QTL mapping and GWAS revealed several overlapping genomic regions associated with productivity-related traits. The most stable loci were located on chromosomes 2D, 4A, 5A, 5B, 6A, 6B, and 7A. From these regions, 16 SNPs were selected for KASP primer design.

The developed KASP markers were validated using a panel of 296 spring common wheat varieties (Panel 2). Among them, 10 markers were successfully amplified and were polymorphic in the tested population. The greatest number of significant associations was detected for three KASP markers, designed for SNPs wsnp_Ex_c16175_24619793 on chromosome 4A, wsnp_Ex_c2171_4072995 on chromosome 5A, and BS00034554_51 on chromosome 6B. These markers demonstrated stable associations with TGW across all three years, while their associations with other traits were less consistent. In particular, wsnp_Ex_c16175_24619793 was additionally associated with GWE; wsnp_Ex_c2171_4072995 showed associations with GWE, GNE, and Yield in individual years; and BS00034554_51 was associated not only with TGW, but also with GWE and Yield. Moreover, the validation panel showed that TGW-associated markers were not consistently associated with yield in all years, while BS00023055_51 was associated with yield but not with TGW, further supporting the complex and environment-dependent relationship between TGW and grain yield.

## 4. Materials and Methods

### 4.1. Plant Material

The following plant material was used in this study (Table 5):

(1) Two F_2_ mapping populations were developed from crosses between the spring bread wheat cultivars Obskaya 2 × Novosibirskaya 15 (Ob2 × N15) and Ob2 × T15 (Ob2 × T15). The cultivar Obskaya 2 belongs to the mid-maturing group and is characterized by high yield potential and good bread-making quality. Novosibirskaya 15 is an early-maturing cultivar with high grain and baking quality and is classified as a strong wheat [55,56]. Tulun 15 is also an early-maturing cultivar with high grain quality but lower yield compared to Obskaya 2. Hybridization, self-pollination of F1 plants, and the production of F_2_ populations were carried out under greenhouse conditions at the Federal Research Center Institute of Cytology and Genetics SB RAS. Field phenotyping was subsequently performed on F_2:3_ families derived from individual F_2_ plants of these populations over two growing seasons (2017–2018) in the Novosibirsk region (Field 2: Novosibirsk region, 54.914070° N, 82.975379° E).

(2) A collection of 92 spring bread wheat (*Triticum aestivum* L.) varieties adapted to the environmental conditions of the Siberian region (Panel 1). The varieties, developed between 1963 and 2008, originated from breeding centers located in seven regions: Samara region (Samarskii NIISKH), Altai Krai (Altaiskii NIIZIS), Novosibirsk region (SibNIIRS), Krasnoyarsk region (Krasnoyarskii NIISKH), Kemerovo region (Kemerovskii NIISKH), Tyumen region (NIISKH Severnogo Zauralya), and Omsk region (Sibirskii NIISKH). A detailed description of this collection has been published previously [56]. Seeds of the varieties are maintained in the collection of the Institute of Cytology and Genetics SB RAS (ICG SB RAS; https://ckp.icgen.ru/plants/fond/ accessed on 29 April 2026). Phenotypic evaluation was conducted under field conditions in the Novosibirsk region during the 2005–2014 (Field 1: Novosibirsk region, 54.884987° N, 82.899519° E) and 2017–2018 growing seasons (Field 2: Novosibirsk region, 54.914070° N, 82.975379° E).

(3) A validation panel consisting of 296 spring bread wheat accessions was assembled based on genetic resources from the Federal Research Center “Nemchinovka” (Moscow region, Odintsovo) and the Institute of Cytology and Genetics SB RAS (Novosibirsk) (Panel 2, Table S12). A detailed description of this panel has been reported previously [57]. Phenotypic evaluation was conducted under field conditions in the Novosibirsk region during the 2022–2024 growing seasons (Field 1: Novosibirsk region, 54.884987° N, 82.899519° E).

### 4.2. Phenotyping

In each growing season, the seeds of F_2:3_ families, panel 1 and panel 2 varieties were sown in the second half of May in 2 repetitions on plots of 2 m^2^ in accordance with the systematic method of sample location. The soil in both fields was leached chernozem. The humus thickness varied from 40 to 60 cm, and the humus content was 4.2%. The soil was slightly acidic (pH 6.7–6.8), with its nitrogen content being 0.34%, total phosphorus-0.30% and potassium-0.13%.

Mature plants were harvested in bundles, air-dried, and subsequently used for the assessment of yield component traits. The grain obtained from the two replicates in each year was mixed together for the following analysis of yield traits. The number of grains per main ear, grain weight per main ear, and thousand grain weight were recorded. Structural analysis was performed on 20 plants per accession. In the case of the ten-year evaluation of traits in Collection 1, grain yield was also assessed. Yield (centners per hectare) was calculated based on grain weight obtained from plots of 2 m^2^. For interpretation, the following conversion can be applied: 1 c/ha = 0.1 t/ha = 100 kg/ha.

Weather data, including monthly averages of daily air temperature and precipitation, were obtained from a meteorological station located in the Novosibirsk region (54.90° N, 82.95° E; altitude 131 m) via the online resource http://www.pogodaiklimat.ru (accessed on 29 April 2026). The environmental conditions during the experimental period were characterized by substantial variation both within individual growing seasons and across years. Total precipitation during the growing season was below average in 2008 (166.8 mm), 2010 (135.9 mm), 2011 (153.5 mm), 2012 (102.7 mm), and 2014 (176 mm), indicating periods of insufficient moisture supply. An extremely severe drought was recorded in July 2012, when only 3.7 mm of precipitation was observed, corresponding to 6.1% of the long-term mean for that period. In contrast, the weather conditions in 2005 and 2009 were closest to the long-term seasonal averages in terms of both temperature and precipitation.

In 2022, moderate average daily temperatures (15.4–18.9 °C) were observed, in 2023, higher temperatures were recorded in June–July (up to 21.6 °C), in 2024, the temperature regime was comparable to that of 2023 (11.3–21.6 °C). Precipitation in 2022 was characterized by dry conditions at the beginning and end of the period, 2023 by contrasting precipitation patterns, and 2024 by excessive moisture throughout the growing season.

### 4.3. DNA Extraction and SNP Genotyping

DNA was extracted following the modified sodium bisulfite protocol described in [58]. DNA purification for SNP genotyping was performed using a Bio-Silica Kit for DNA Purification from Reaction Mixtures according to the manufacturer’s protocol. The DNA was then quantified using the NanoDrop M2000 (Thermo Fisher Scientific, Waltham, MA, USA).

The panel 1 of spring bread wheat cultivars was genotyped using the Illumina Infinium 15K Wheat SNP array by TraitGenetics-Section of SGS Institute Fresenius GmbH (Gatersleben, Germany) in 2016 [59]. The F_2_ mapping populations were genotyped using the same Illumina Infinium 15K Wheat SNP array (San Diego, CA, USA), supplemented with an additional 4090 markers from the Affymetrix Axiom platform (Santa Clara, CA, USA) in 2017.

### 4.4. Genetic Linkage Maps and QTL Analysis

Genotyping data obtained for the F_2_ mapping populations of bread wheat derived from the crosses Ob2 × N15 (92 lines) and Ob2 × T15 (84 lines) were used to construct genetic linkage maps using the software MultiPoint UltraDense v 4.1 [60]. During map construction, markers with more than 25 missings and segregation distortion exceeding χ^2^ = 42 were removed. The minimum size of a co-segregating marker group (i.e., linked markers located at the same map position) was set to two markers. Marker clustering was performed using a recombination fraction threshold of rf = 1.5. Marker ordering within clusters was carried out using the GES (guided evolutionary strategy) algorithm with jackknife resampling. To obtain stable maps, a monotonicity control procedure was applied, followed by the removal of outlier markers and the sequential elimination of destabilizing markers.

Using the constructed genetic maps together with phenotypic data, QTL mapping was performed for thousand grain weight (TKW), grain number per spike, and grain weight per spike. QTL localization was carried out using the MultiQTL v 2.6 software based on the MIM (Multiple Interval Mapping) algorithm with a significance threshold of 99.9%. Visualization of the genetic maps was performed using MapChart version 2.3 [61].

### 4.5. Genome-Wide Association Study

Genome-wide association analysis was performed using genotyping data from panel 1 together with phenotypic data for TGW, GNE, and GWE collected over two years of field trials, and data on grain yield and grain weight per spike obtained over ten years of field trials. Only polymorphic SNPs were selected for subsequent analysis. The genotyping data were filtered by minor allele frequency (MAF > 0.05) and by missing data less than 20% of population. Linear mixed models (MLM) implemented in the software TASSEL v.5.2.50 [62] were used to detect marker-trait associations. The model accounted for population structure (Q) as well as the kinship matrix (K). Population structure matrix was calculated using the R package LEA v 3.10.2 based on a STRUCTURE-like inference algorithm [63]. A kinship matrix was calculated using the “Centered_IBS” method with default settings in TASSEL v.5.2.50.

### 4.6. KASP Validation

Selected single nucleotide polymorphisms (SNPs) identified in this study were validated using the Kompetitive Allele-Specific PCR (KASP) genotyping assay. Primers for the chosen SNPs were designed and synthesized by LGC Genomics (Teddington, UK). PCR reactions were performed using the KASP master mix supplied by LGC Genomics, following the manufacturer’s standard protocol.

Reactions were set up in 96-well plates with a total volume of 10 µL per reaction, containing 5 µL of KASP master mix, 0.14 µL of primer mix (allele-specific and common primers), and 100 ng of genomic DNA. PCR amplification was conducted on an ABI 7500 Real-Time PCR System (Applied Biosystems, Waltham, MA, USA) using the standard cycling program: initial denaturation at 94 °C for 15 min, followed by 10 touchdown cycles of 94 °C for 20 s and 61–55 °C for 60 s (dropping 0.6 °C per cycle), then 26 cycles of 94 °C for 20 s and 55 °C for 60 s.

Allelic discrimination and genotype calling were performed using the Design & Analysis Software Release 2.8.0 (Thermo Fisher Scientific). Reference genotypes with known alleles were included in each plate as positive controls to ensure assay accuracy and reproducibility. SNPs that showed clear clustering and consistent genotyping results across replicates were considered successfully validated.

### 4.7. Statistical Analysis

All statistical analyses were performed in R v 4.3 software. Normality of trait distributions was assessed using the Shapiro–Wilk test, and data distribution was additionally visualized using histograms. Broad-sense heritability (H^2^) was estimated from the variance components calculated using the R package lme4 v 2.0 using the “Standard” broad-sense heritability method [64]. Pearson correlation coefficients among traits and across years were calculated using pairwise complete observations, and significance levels of correlations were determined using the rcorr function from the Hmisc v 5.2 package. Pairwise relationships among traits were additionally explored using scatterplot matrices generated with the GGally v 2.4.0 package.

Marker-trait associations were evaluated using one-way models with marker genotype treated as a fixed factor. For each trait-marker combination, model assumptions were verified prior to hypothesis testing. Normality of residuals was assessed using the Shapiro–Wilk test. Homogeneity of variances was tested using Levene’s test.

Depending on the assumption diagnostics, the following statistical procedures were applied: one-way ANOVA was used when residuals were normally distributed and variances were homogeneous; Welch’s ANOVA was applied when residuals were normally distributed but variance heterogeneity was detected; and the Kruskal–Wallis test was used when residuals significantly deviated from normality. Effect sizes were calculated to estimate the magnitude of marker effects. Post hoc comparisons were performed for all models: Tukey’s HSD test after classical ANOVA; Games-Howell test after Welch ANOVA; Dunn’s test after Kruskal–Wallis. To control for multiple testing across all marker–trait combinations, false discovery rate (FDR) correction was applied using the Benjamini–Hochberg method.

## 5. Conclusions

In this study, the genetic architecture of major wheat productivity-related traits, including grain number per ear, grain weight per ear, thousand grain weight, and yield, was investigated in Russian spring wheat cultivars and mapping populations adapted to diverse agro-climatic regions of Russia. By combining QTL mapping and GWAS approaches across multiple experimental panels and years of phenotyping, several stable genomic regions consistently associated with productivity-related traits were identified.

The most reproducible loci were detected on chromosomes 2D, 4A, 5A, 5B, 6A, 6B, and 7A. In particular, loci on chromosomes 2D, 4A, 5A, 5B, and 6A were associated with all four studied traits (Yield, GNE, TGW, and GWE), while loci on chromosomes 6B and 7A were associated with Yield, GNE, and GWE. The overlap between QTL mapping and GWAS results supports the robustness and stability of these genomic regions across different genetic backgrounds and environmental conditions.

Validation of the developed KASP markers revealed stable associations primarily with TGW, as well as additional associations with other agronomic traits in individual years. Based on these results, the markers wsnp_Ex_c16175_24619793, wsnp_Ex_c2171_4072995, and BS00034554_51 may represent promising tools for marker-assisted selection of favorable grain-related traits. Although TGW showed a positive association with grain yield in the validation panel, this relationship was moderate, while TGW was weakly negatively correlated with GNE. These results indicate that TGW should not be considered a direct proxy for grain yield, but rather one of several interacting components contributing to wheat productivity. Therefore, the identified TGW-associated loci and KASP markers require further validation to confirm their direct contribution to grain yield improvement across environments and genetic backgrounds.

## Figures and Tables

**Table 1:** Results of QTL analysis in the Ob2 × N15 population. Chr—chromosome; d—additive effect of the allele; PA—parent contributing the allele with a positive effect (O—Obskaya 2, N—Novosibirskaya 15).

Trait	Chr	Position on Genetic Linkage Map, cM	Flanking Markers	Position on RefSeq v1.1	LOD	d	PA
2017 year							
GNE	4A	100.9–122.2	wsnp_Ex_c539_1072859-RAC875_c42756_168	467,297,790–597,688,211	3.61	2.31	O
GNE	5A	138.2–142.2	Tdurum_contig71499_211-Excalibur_c79344_151	570,933,960–575,700,138	4.30	−1.55	N
GNE	7BS	0–5.07	Ex_c101666_634-BS00010616_51	24,060,479–63,569,924	5.77	1.79	O
GWE	6D	0–22.02	CAP7_c1467_220-Ex_c5168_849	461,925,085–462,464,820	4.16	0.14	O
GWE	3A	21.9–47.1	BS00064039_51-wsnp_Ku_c44716_51926415	572,321,365–600,007,677	3.05	0.12	O
TGW	2D	2.2–56.6	BobWhite_rep_c51612_864-RAC875_rep_c69194_867	77,132,627–580,971,442	3.20	2.00	O
2018 year							
GNE	4A	32.2–55.4	RAC875_c35819_165-Tdurum_contig15586_563	684,514,599–649,808,983	6.80	3.88	O
GNE	4A	69.6–108.6	RFL_Contig4336_184-Excalibur_c32735_603	600,904,720–584,799,733	6.47	3.77	O
GNE	7A	66. 8–85.3	Ra_c9427_300-wsnp_Ku_c6065_10682531	78,434,149–109,843,568	6.29	−3.65	N
GNE	5B	0–5.12	Tdurum_contig25432_1377-RAC875_c8661_692	19,439,296–26,951,039	4.74	−2.19	N
GNE	6B	74.01–91.04	Tdurum_contig569_263-wsnp_Ku_c43368_50890819	679,243,223–704,793,445	4.12	0.44	O
GNE	7BS	0–5.02	Ex_c101666_634-BS00010616_51	24,060,479–63,569,924	3.47	2.36	O
GWE	2A	91.9–99.6	wsnp_Ex_c10751_17505459-BobWhite_c16923_64	716,903,314–721,562,548	7.06	−0.01	N
GWE	4A	95.7–108.6	RAC875_c94519_448-Excalibur_c32735_603	600,904,720–584,799,733	5.60	0.10	O
GWE	4A	111.4–122.2	BS00010339_51-RAC875_c42756_168	583,953,406–467,297,790	6.66	0.11	O
GWE	2B	93.2–128.4	IAAV717-wsnp_Ex_c14595_22634031	158,618,802–592,585,252	7.12	−0.01	N
GWE	5B	0–5.1	Tdurum_contig25432_1377-RAC875_c8661_692	19,439,296–26,951,039	5.40	−0.06	N
GWE	7B	0–12.9	Ex_c101666_634-GENE-4333_211	24,060,479–113,626,728	4.71	0.10	O
GWE	5D	4.3–38. 5	Kukri_rep_c104865_57-tplb0030a05_2386	544,614,710–562,726,525	6.33	0.14	O
TGW	5A	220.1–225.9	BS00021968_51-Excalibur_c1954_930	677,631,865–680,503,202	4.09	3.56	O

**Table 2:** Results of QTL analysis in the Ob2 × T15 population. Chr—chromosome; d—additive effect of the allele; PA—parent contributing the allele with a positive effect (O—Obskaya 2, T—Tulun 15).

Trait	Chr	Position on Genetic Linkage Map, cM	Flanking Markers	Position on RefSeq v 1.1	Max LOD	d	PA
2017 year							
GNE	4A	96.9–97.5	RAC875_c95150_286-BobWhite_c10610_1096	684,968,700–686,382,538	5.09	4.39	O
GNE	2D	39.8–95.3	wsnp_CAP12_c1503_764765-Excalibur_c30328_713	35,683,548–596,912,244	5.82	4.53	O
GNE	5D	2.4–4.8	Kukri_rep_c73094_348-wsnp_Ex_rep_c67164_65655648	100,931,464–278,625,785	3.97	−3.04	T
GNE	6D	1.3–16.39	Excalibur_c21688_724-BS00003697_51	26,255,099–464,943,892	2.48	−2.74	T
GWE	4A	90.2–109	RFL_Contig4336_184-D_F5XZDLF02G9H4M_286	640,249,419–686,382,538	7.29	0.18	O
GWE	7A	33.2–75.4	AX-94525876-BS00102773_51	31,875,862–65,476,438	4.43	−0.12	T
GWE	2D	39.8–95.3	wsnp_CAP12_c1503_764765-Excalibur_c30328_713	35,683,548–596,912,244	11.91	0.23	O
TGW	2A	36.7–75.8	wsnp_Ex_rep_c101526_86881496-Excalibur_c41125_360	71,421,116–714,207,690	12.32	3.30	O
TGW	4A	120.9–150.3	BS00022395_51-RFL_Contig3621_947	693,278,257–742,335,547	8.97	1.50	O
TGW	5A	134.6–186.1	AX-94422048-CAP7_c4064_162	622,380,065–689,916,978	11.19	2.57	O
TGW	2B	20.6–34.9	BS00081871_51-AX-94391750	12,076,560–32,963,435	7.97	−1.52	T
TGW	4B	87.9–91.3	Kukri_c18722_56-Kukri_c33550_601	14,109,759–656,902,757	7.29	2.03	O
TGW	2D	54.4–65.4	AX-95124335-wsnp_BE444144D_Ta_1_1	52,232,863–181,596,928	6.05	2.79	O
TGW	4D	0–2.4	Kukri_c64744_698-BS00099053_51	3,395,840–3,612,636	5.71	2.19	O
TGW	5D	0–4.8	RAC875_rep_c72023_267-wsnp_Ex_rep_c67164_65655648	100,931,464–341,565,539	5.93	0.57	O
2018 year							
GNE	2A	44.7–50.1	Tdurum_contig27887_55-BobWhite_c28819_733	156,366,698–564,620,629	5.55	−2.52	T
GNE	2D	69.0–97.1	BS00021718_51-tplb0021e03_713	83,854,119–609,776,007	7.37	3.53	O
GWE	2D	28.2–54.4	BS00022276_51-AX-95124335	29,454,272–52,232,863	5.72	0.22	O
GWE	2D	69.0–95.3	BS00021718_51-Excalibur_c30328_713	83,854,119–596,912,244	5.44	0.25	O
TGW	2A	36.7–60.4	wsnp_Ex_rep_c101526_86881496-RAC875_rep_c113120_326	71,421,116–675,938,530	5.54	2.86	O
TGW	5A	106.8–129.7	JD_c15758_288-Ex_c6479_750	595,373,154–613,703,560	5.67	2.32	O
TGW	6A	51.01–80.4	Kukri_rep_c104648_106-Excalibur_c41490_397	33,954,815–531,522,393	6.75	3.61	O
TGW	2D	28.2–54.4	BS00022276_51-AX-95124335	29,454,272–52,232,863	6.61	3.67	O
TGW	3A	131.9–140.3	BS00022459_51-BS00081475_51	697,454,068–701,891,172	4.30	0.20	O

**Table 3:** Integrated comparison of QTL and GWAS results for yield and its components in wheat. QTLs were identified in two biparental populations, QTL ON (Obskaya 2 × Novosibirskaya 15) and QTL OT (Ob2 × T15), while GWAS was performed using a diversity panel based on long-term phenotypic data (10 years; Yield and grain weight per ear, GWE) and short-term data (2017–2018; grain number per ear, GNE; grain weight per ear, GWE; thousand grain weight, TGW). Genomic regions are presented as approximate physical intervals (Mb) based on the reference genome RefSeq v 1.1. Overlapping loci across datasets are indicated, and associated traits are summarized for each region.

Chr	Region (Mb, RefSeq v 1.1)	QTL ON	QTL OT	GWAS (10 Years)	GWAS (Two Years)	Traits
1B	15–30; 360–430; 660–690	–	–	Yield, GWE	GNE, GWE	Yield, GWE, GNE
2A	680–712	GWE	TGW	GWE	GWE	GWE, TGW
2D	50–160	TGW	GNE, GWE, TGW	Yield	TGW	Yield, TGW, GNE, GWE
3A	730–740	GWE	GWE	GWE	GNE, TGW, GWE	GNE, TGW, GWE
4A	580–620	GNE, GWE	GNE, GWE, TGW	Yield, GWE	TGW, GWE	Yield, GNE, TGW, GWE
5A	480–680	TGW	TGW	Yield, GWE	GNE, TGW, GWE	Yield, GNE, TGW, GWE
5B	0–5; ~540; ~680	GNE, GWE	GNE, GWE, TGW	Yield, GWE	TGW, GWE, GNE	Yield, GNE, TGW, GWE
6A	600–615	–	TGW	Yield	GNE, GWE	Yield, GNE, TGW, GWE
6B	150–230; 650–710	GNE	–	Yield, GWE	GNE, GWE	Yield, GNE, GWE
7A	60–100; ~640	GNE	GWE	Yield, GWE	GNE, GWE	Yield, GNE, GWE

**Table 4:** Summary of associations detected for the three most informative KASP markers in the validation panel (Panel 2). Full marker-trait association results are provided in Tables S10 and S11. *, *p* < 0.05; **, *p* < 0.01; ***, *p* < 0.001.

KASP Marker	Chr	Most Stable Association	Additional Associations Detected in Individual Years
wsnp_Ex_c16175_24619793	4A	TGW: 2022 ***, 2023 ***, 2024 ***, mean ***	GWE: 2022 *, 2024 *, mean **
wsnp_Ex_c2171_4072995	5A	TGW: 2022 ***, 2023 ***, 2024 ***, mean ***	GWE: 2024 **; GNE: 2023 **, 2024 *, mean **; Yield: 2022 *
BS00034554_51	6B	TGW: 2022 ***, 2023 ***, 2024 ***, mean ***	GWE: 2024 *; Yield: 2022 *, 2023 **, mean **

**Table 5:** Summary information about plant collections, used in this study, field and year of their cultivation, measured traits.

Parents	Field	Year	Trait	Short Name of Trait
F_2:3_ Obskaya 2 × Novosibirskaya 15	field 2	2017–2018	Grain number per ear	ON_GNE
			Grain weight per ear	ON_GWE
			Thousand grain weight	ON_TGW
F_2:3_ Obskaya 2 × Tulun 15	field 2	2017–2018	Grain number per ear	OT_GNE
			Grain weight per ear	OT_GWE
			Thousand grain weight	OT_TGW
92 varieties of spring common wheat	field 1	2005–2014	Grain weight per ear	Panel1_GWE_10Y
			Yield (centner/ha)	Panel1_Yield_10Y
	field 2	2017–2018	Grain number per ear	Panel1_GNE_2Y
			Grain weight per ear	Panel1_GWE_2Y
			Thousand grain weight	Panel1_TGW_2Y
296 varieties of spring common wheat	field 1	2022–2024	Grain weight per ear	Panel2_GWE_2Y
			Thousand grain weight	Panel2_TGW_2Y
			Thousand grain weight	Panel2_TGW

## Data Availability

The original contributions presented in this study are included in the article/Supplementary Materials. Further inquiries can be directed to the corresponding author.

## Supplementary Materials

The following supporting information can be downloaded at https://www.mdpi.com/article/10.3390/ijms27115130/s1.

## Abbreviations

The following abbreviations are used in this manuscript:

GWAS	Genome-wide association studies
QTL	Quantitative Trait Loci
MTAs	Marker–trait associations
SNP	Single-nucleotide polymorphism
GNE	Grain number per ear
GWE	Grain weight per ear
TGW	Thousand grain weight
