# Supplementary material for: Identification and Validation of Stable Loci Underlying Productivity-Related Traits in Common Wheat
Source: Int J Mol Sci. 2026 Jun 5;27(11):5130. doi: 10.3390/ijms27115130 (PMC13257091; doi:10.3390/ijms27115130)
Supplement: Supplementary file 1 [file ijms-27-05130-s001.zip › Suppl_Figures_04.06.2026_edt.pptx]

## Slide 1
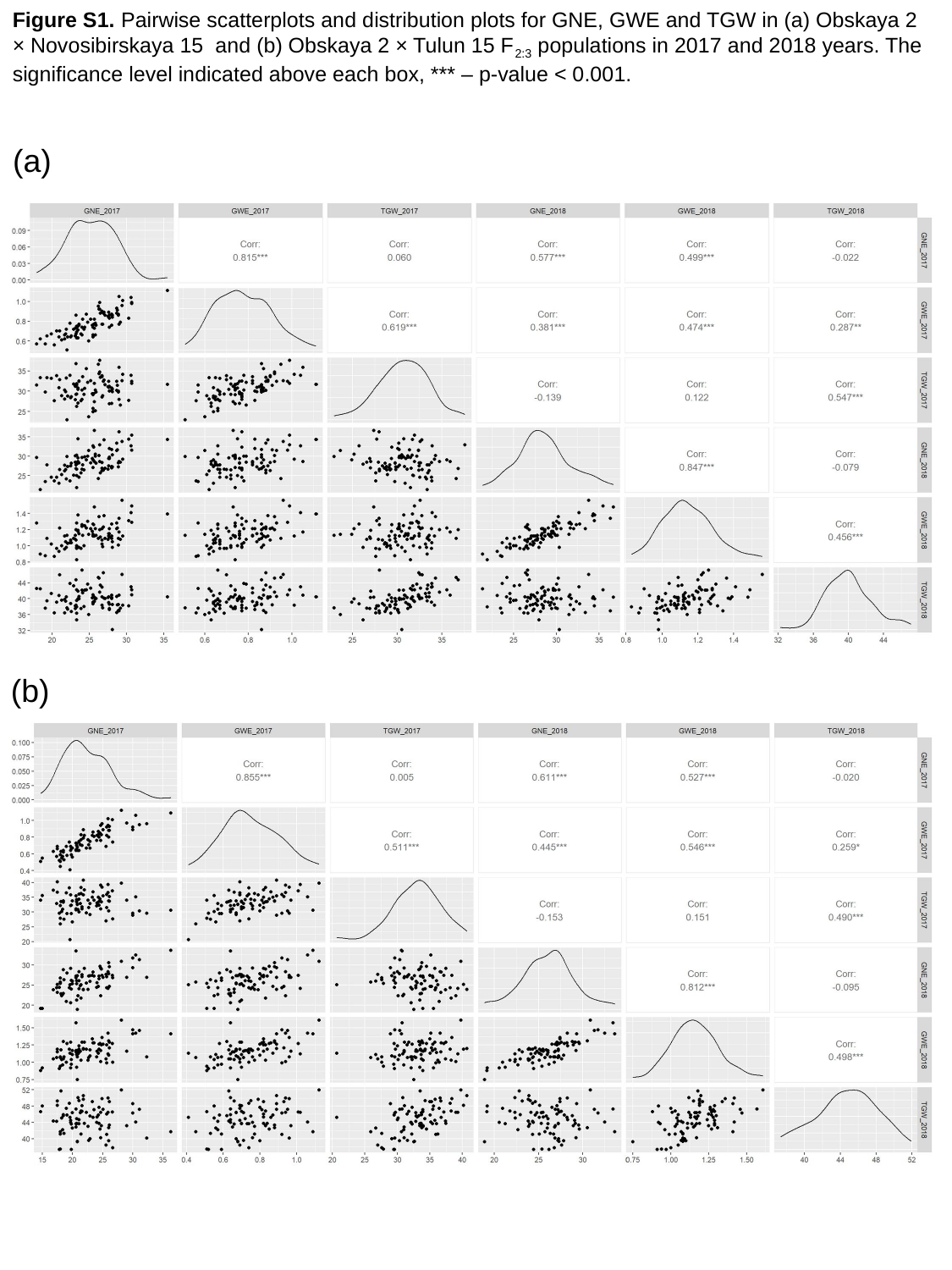

Figure S1. Pairwise scatterplots and distribution plots for GNE, GWE and TGW in (a) Obskaya 2 × Novosibirskaya 15 and (b) Obskaya 2 × Tulun 15 F2:3 populations in 2017 and 2018 years. The significance level indicated above each box, *** – p-value < 0.001.
(a)
(b)

## Slide 2
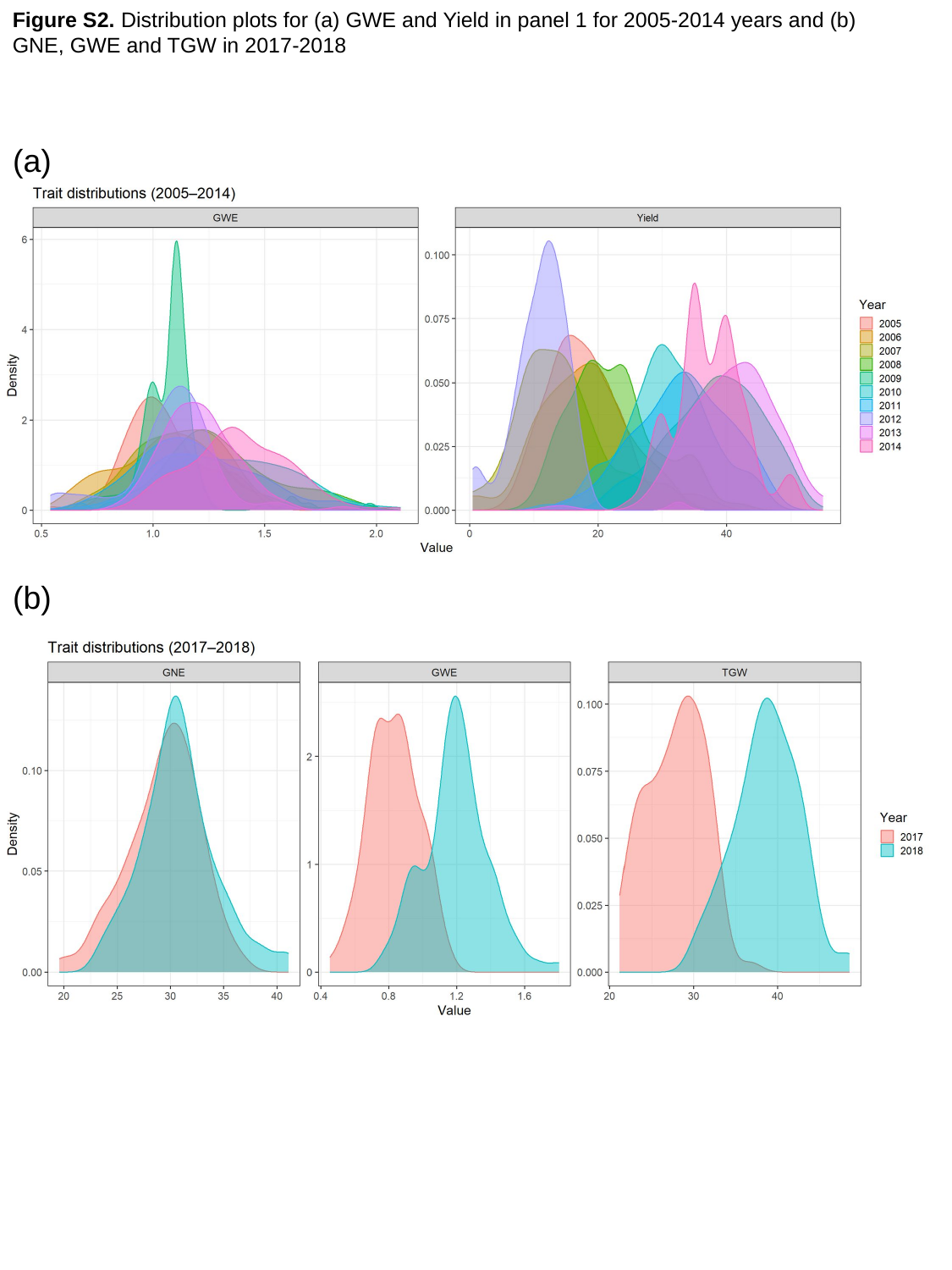

Figure S2. Distribution plots for (a) GWE and Yield in panel 1 for 2005-2014 years and (b)
GNE, GWE and TGW in 2017-2018
(a)
(b)

## Slide 3
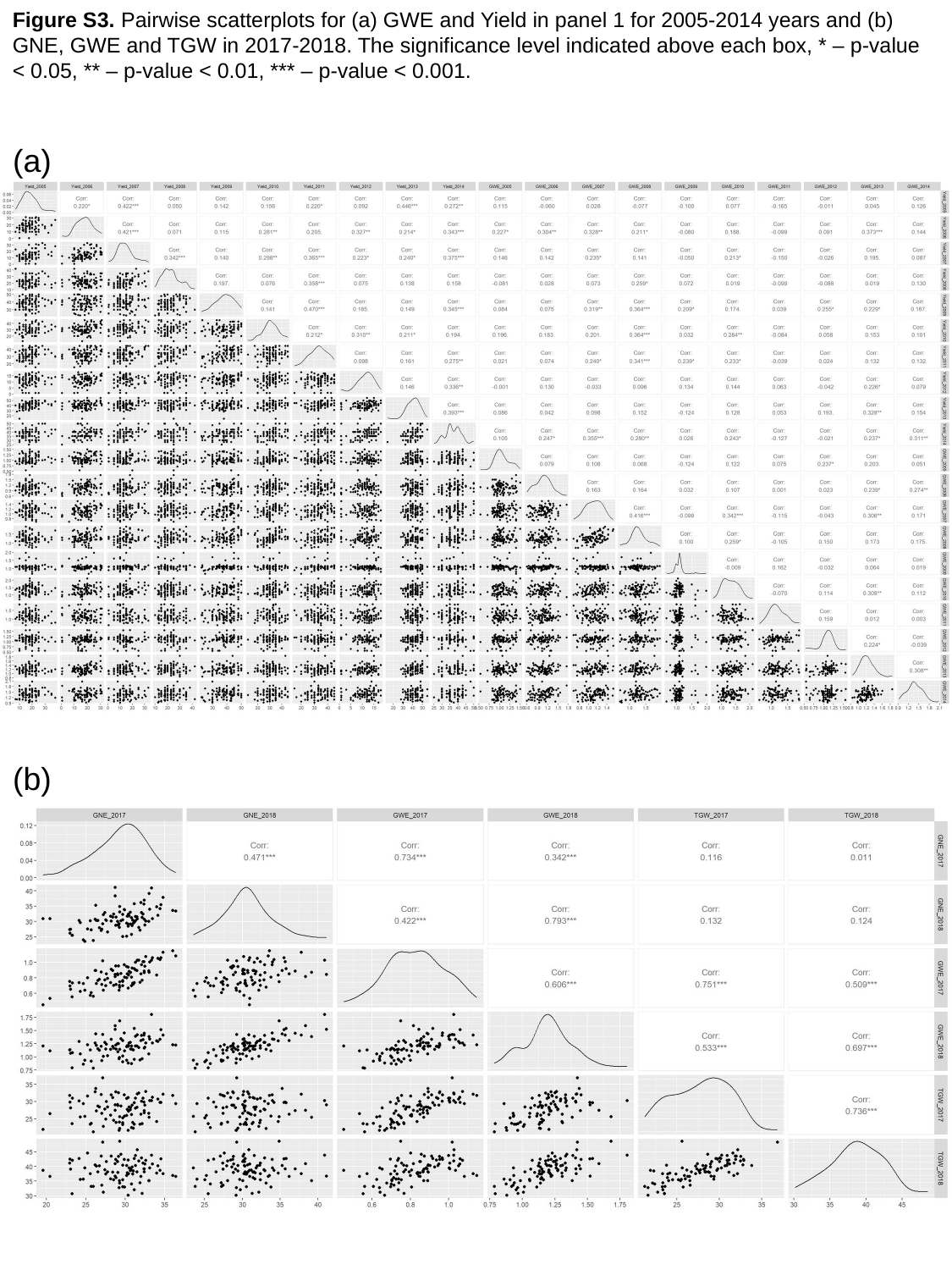

Figure S3. Pairwise scatterplots for (a) GWE and Yield in panel 1 for 2005-2014 years and (b)
GNE, GWE and TGW in 2017-2018. The significance level indicated above each box, * – p-value < 0.05, ** – p-value < 0.01, *** – p-value < 0.001.
(a)
(b)

## Slide 4
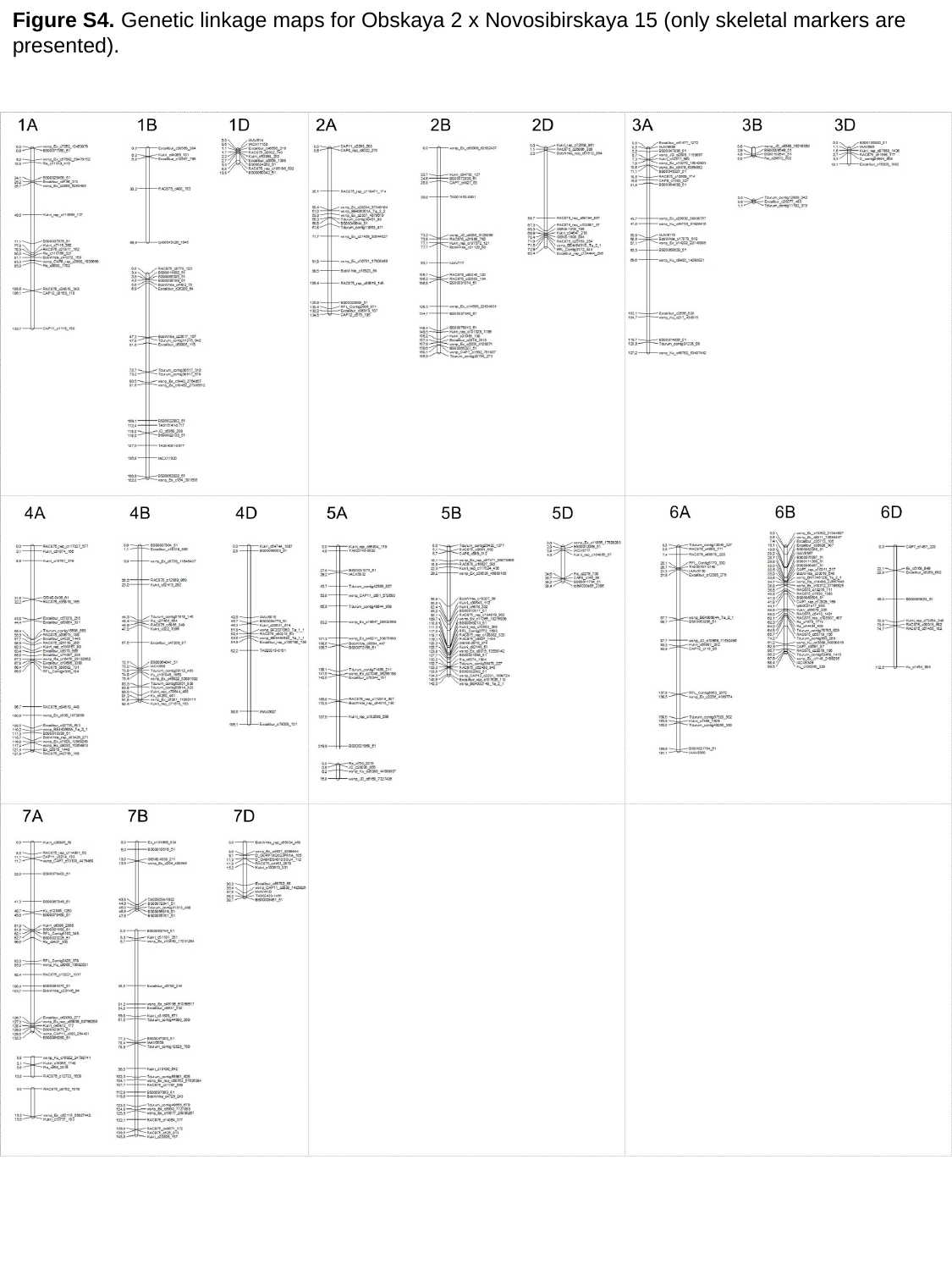

Figure S4. Genetic linkage maps for Obskaya 2 x Novosibirskaya 15 (only skeletal markers are presented).

## Slide 5
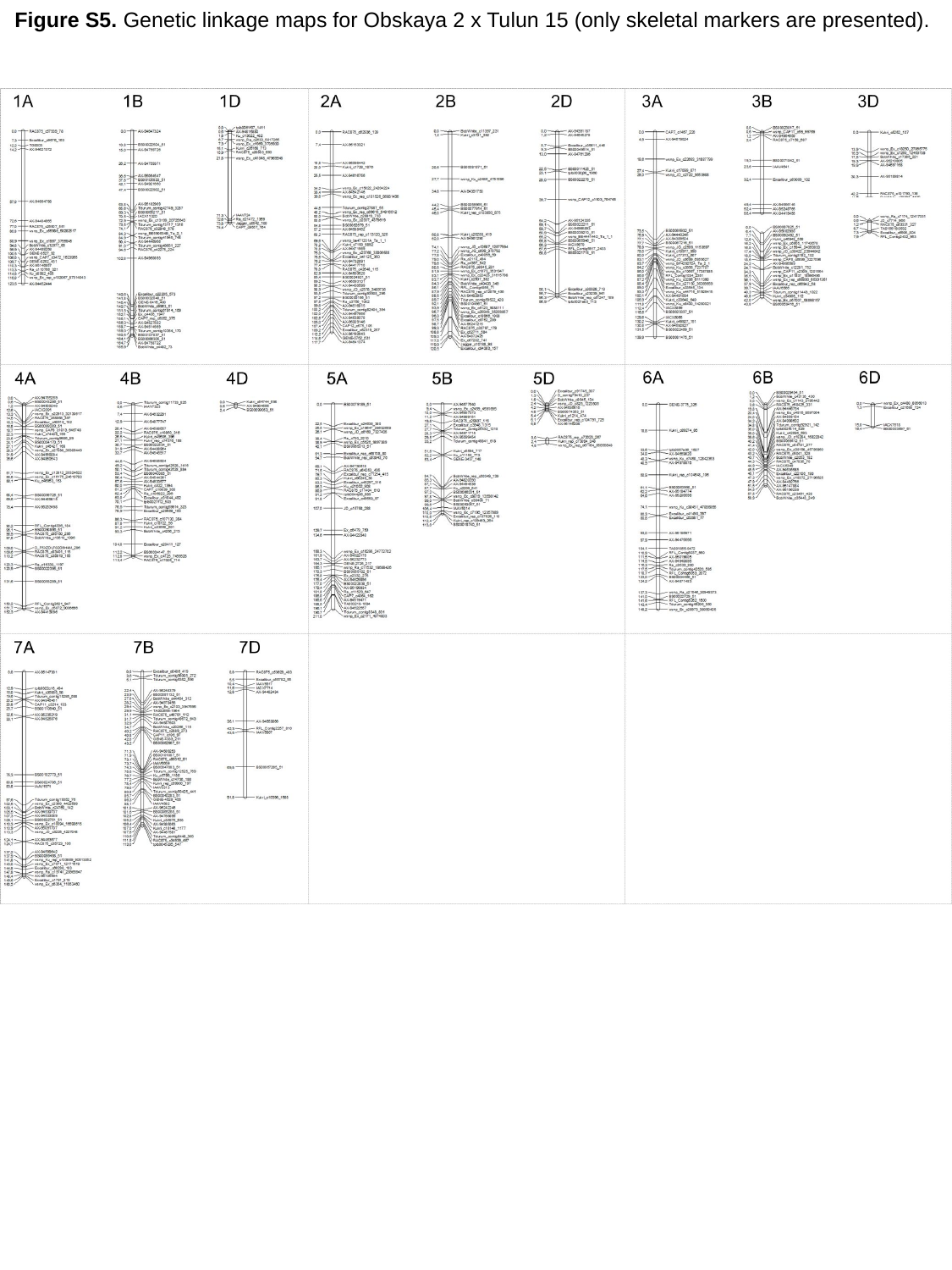

Figure S5. Genetic linkage maps for Obskaya 2 x Tulun 15 (only skeletal markers are presented).

## Slide 6
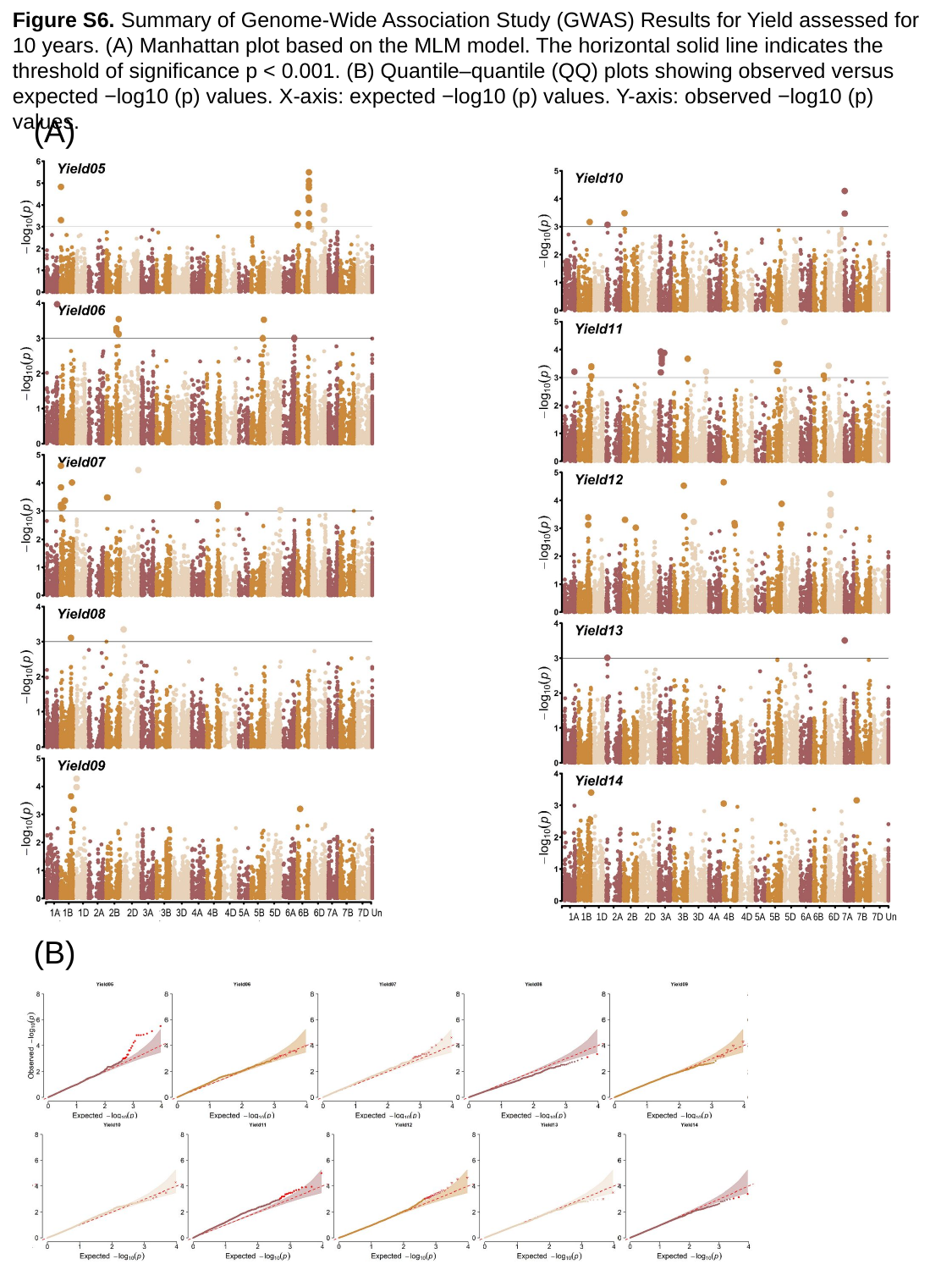

Figure S6. Summary of Genome-Wide Association Study (GWAS) Results for Yield assessed for 10 years. (A) Manhattan plot based on the MLM model. The horizontal solid line indicates the threshold of significance p < 0.001. (B) Quantile–quantile (QQ) plots showing observed versus expected −log10 (p) values. X-axis: expected −log10 (p) values. Y-axis: observed −log10 (p) values.
(A)
1A 1B 1D 2A 2B 2D 3A 3B 3D 4A 4B 4D 5A 5B 5D 6A 6B 6D 7A 7B 7D Un
(B)

## Slide 7
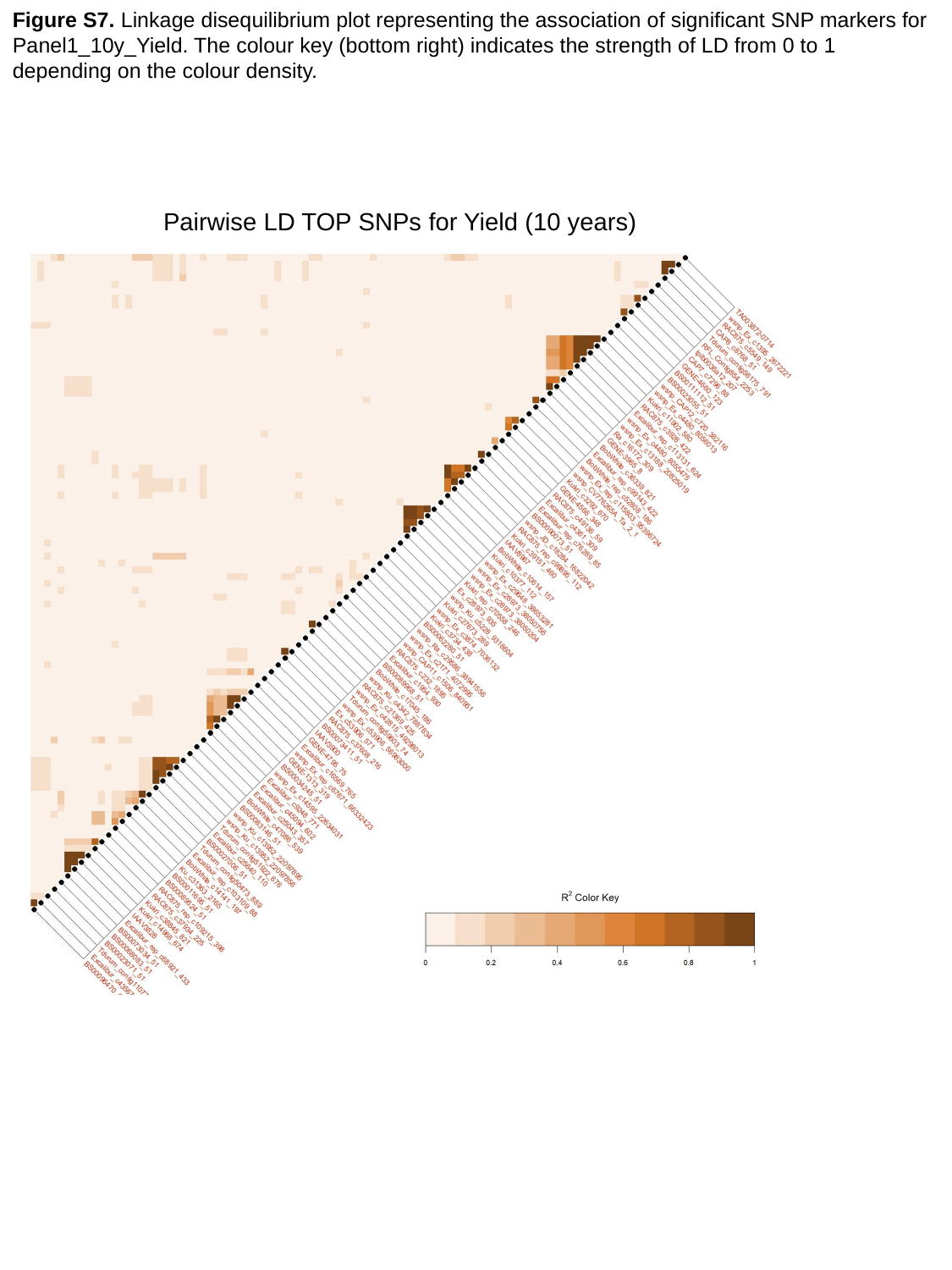

Figure S7. Linkage disequilibrium plot representing the association of significant SNP markers for Panel1_10y_Yield. The colour key (bottom right) indicates the strength of LD from 0 to 1 depending on the colour density.
Pairwise LD TOP SNPs for Yield (10 years)

## Slide 8
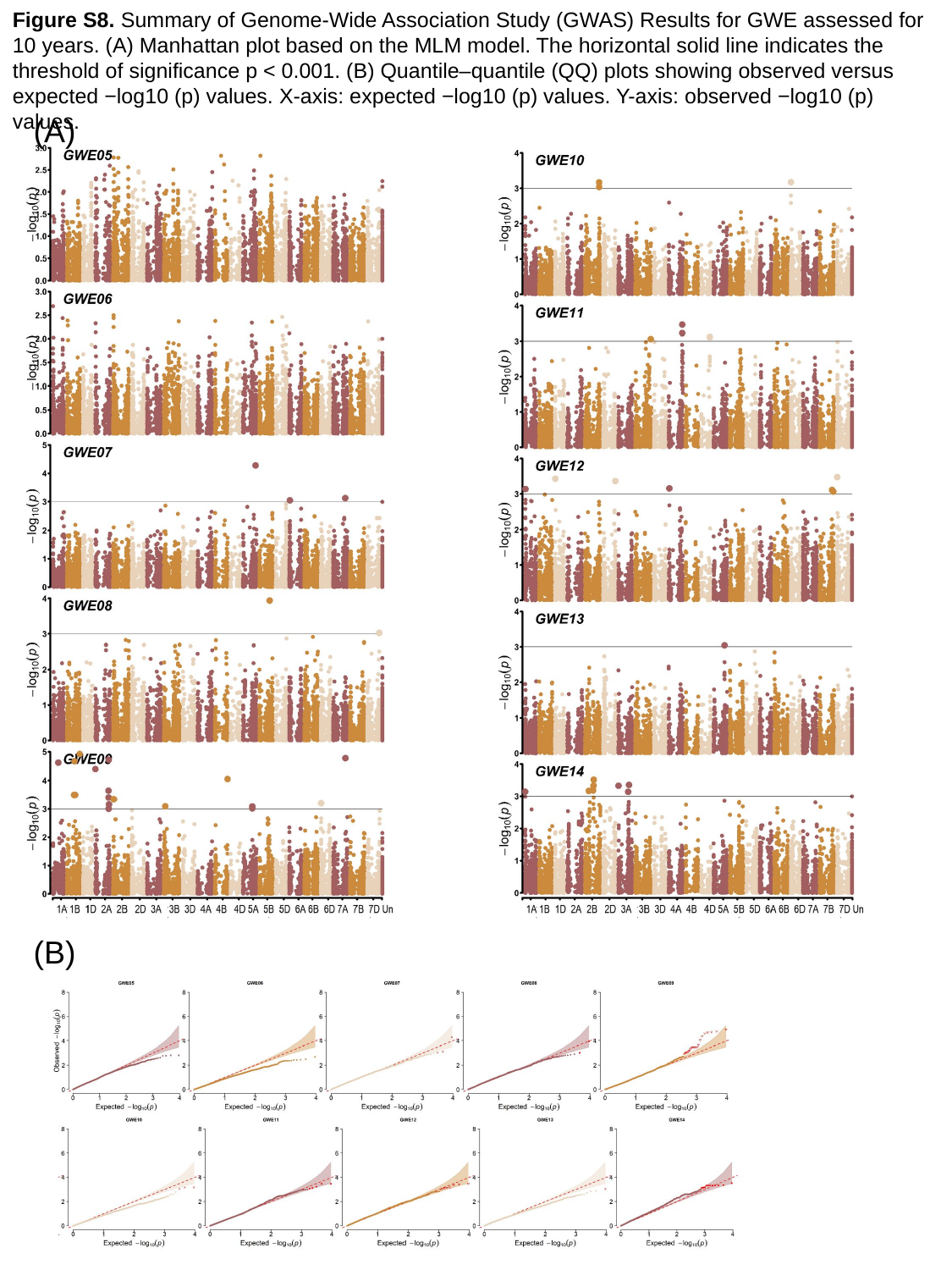

Figure S8. Summary of Genome-Wide Association Study (GWAS) Results for GWE assessed for 10 years. (A) Manhattan plot based on the MLM model. The horizontal solid line indicates the threshold of significance p < 0.001. (B) Quantile–quantile (QQ) plots showing observed versus expected −log10 (p) values. X-axis: expected −log10 (p) values. Y-axis: observed −log10 (p) values.
(A)
(B)

## Slide 9
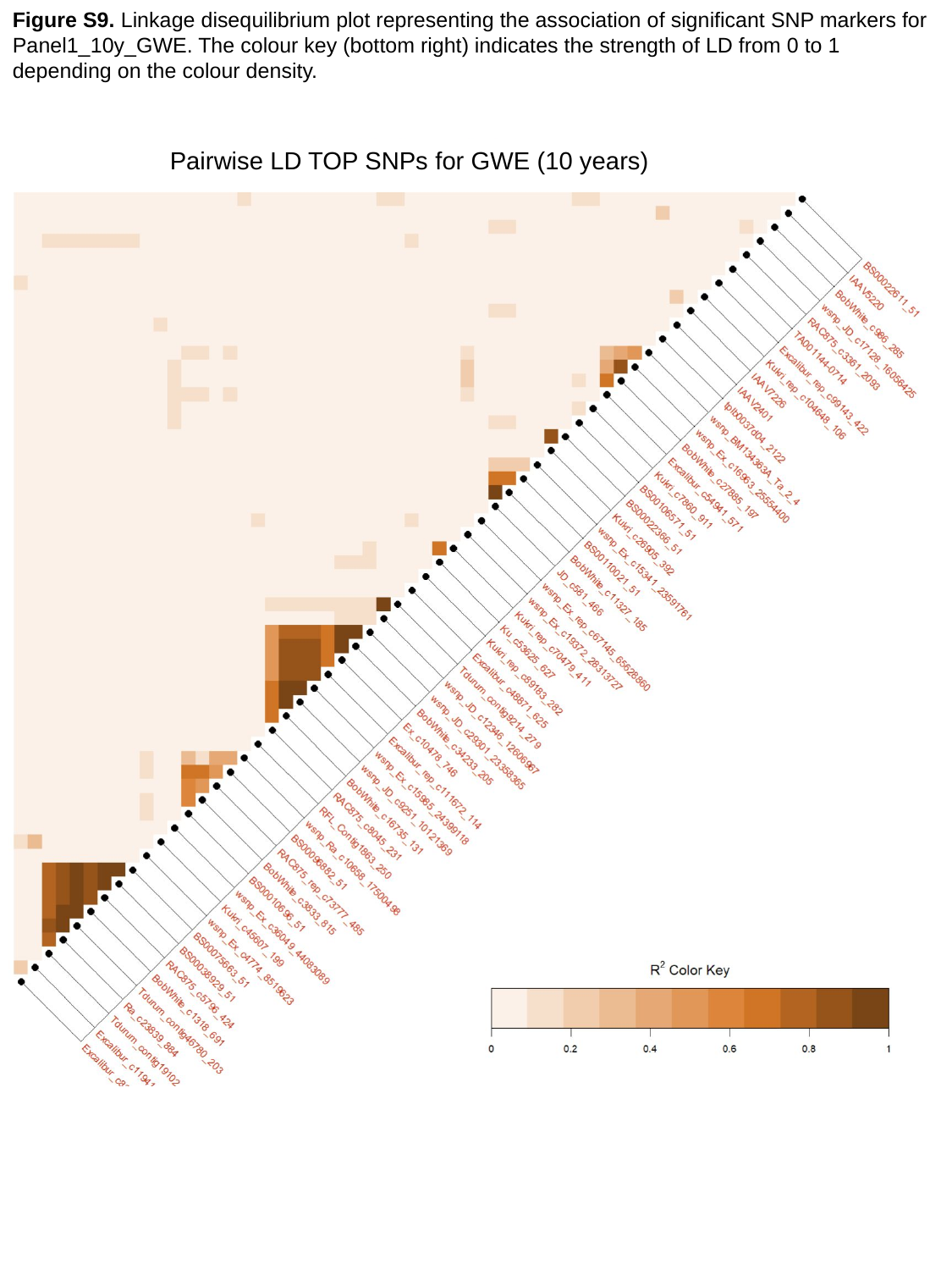

Figure S9. Linkage disequilibrium plot representing the association of significant SNP markers for Panel1_10y_GWE. The colour key (bottom right) indicates the strength of LD from 0 to 1 depending on the colour density.
Pairwise LD TOP SNPs for GWE (10 years)

## Slide 10
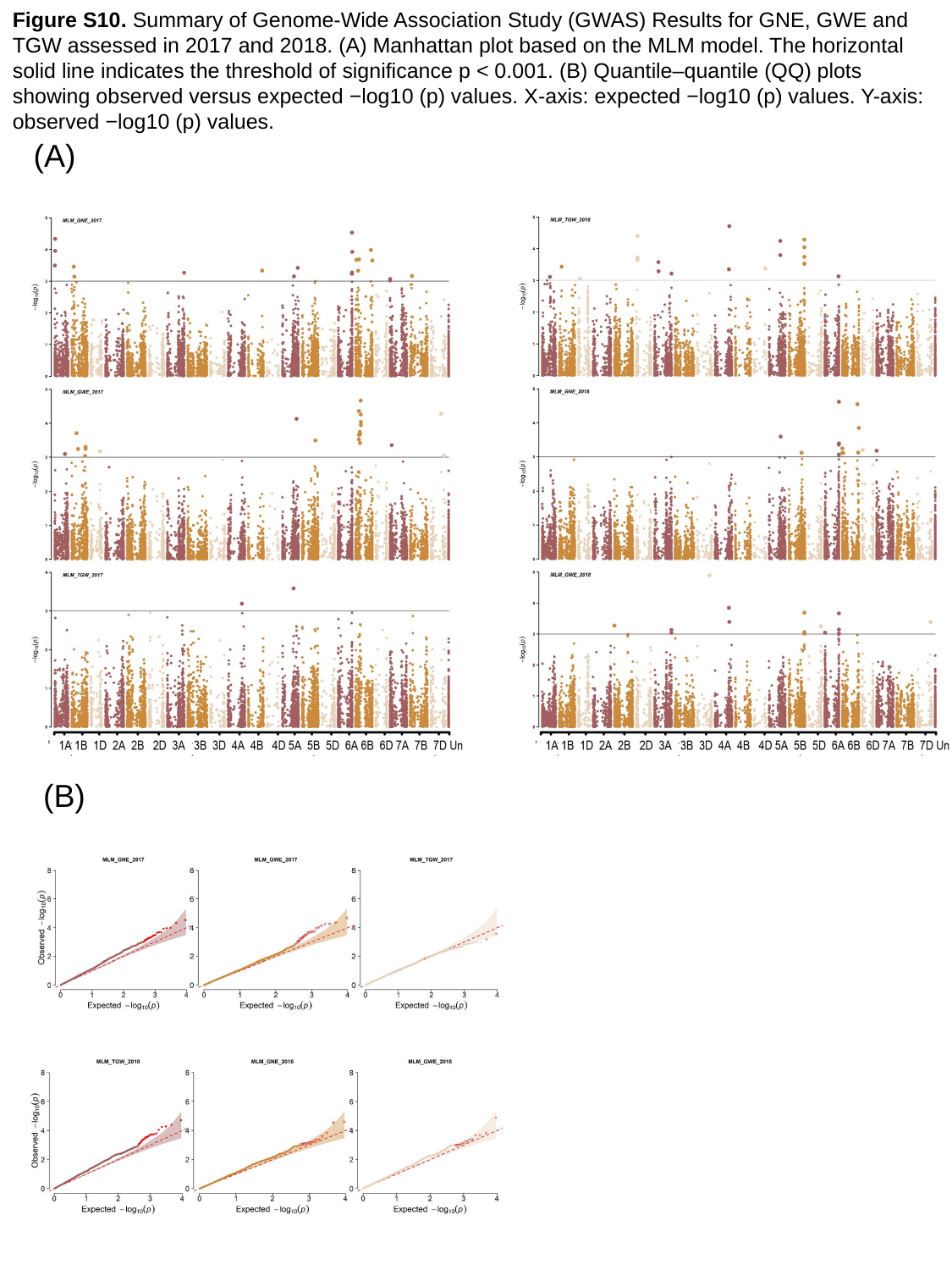

Figure S10. Summary of Genome-Wide Association Study (GWAS) Results for GNE, GWE and TGW assessed in 2017 and 2018. (A) Manhattan plot based on the MLM model. The horizontal solid line indicates the threshold of significance p < 0.001. (B) Quantile–quantile (QQ) plots showing observed versus expected −log10 (p) values. X-axis: expected −log10 (p) values. Y-axis: observed −log10 (p) values.
(A)
(B)

## Slide 11
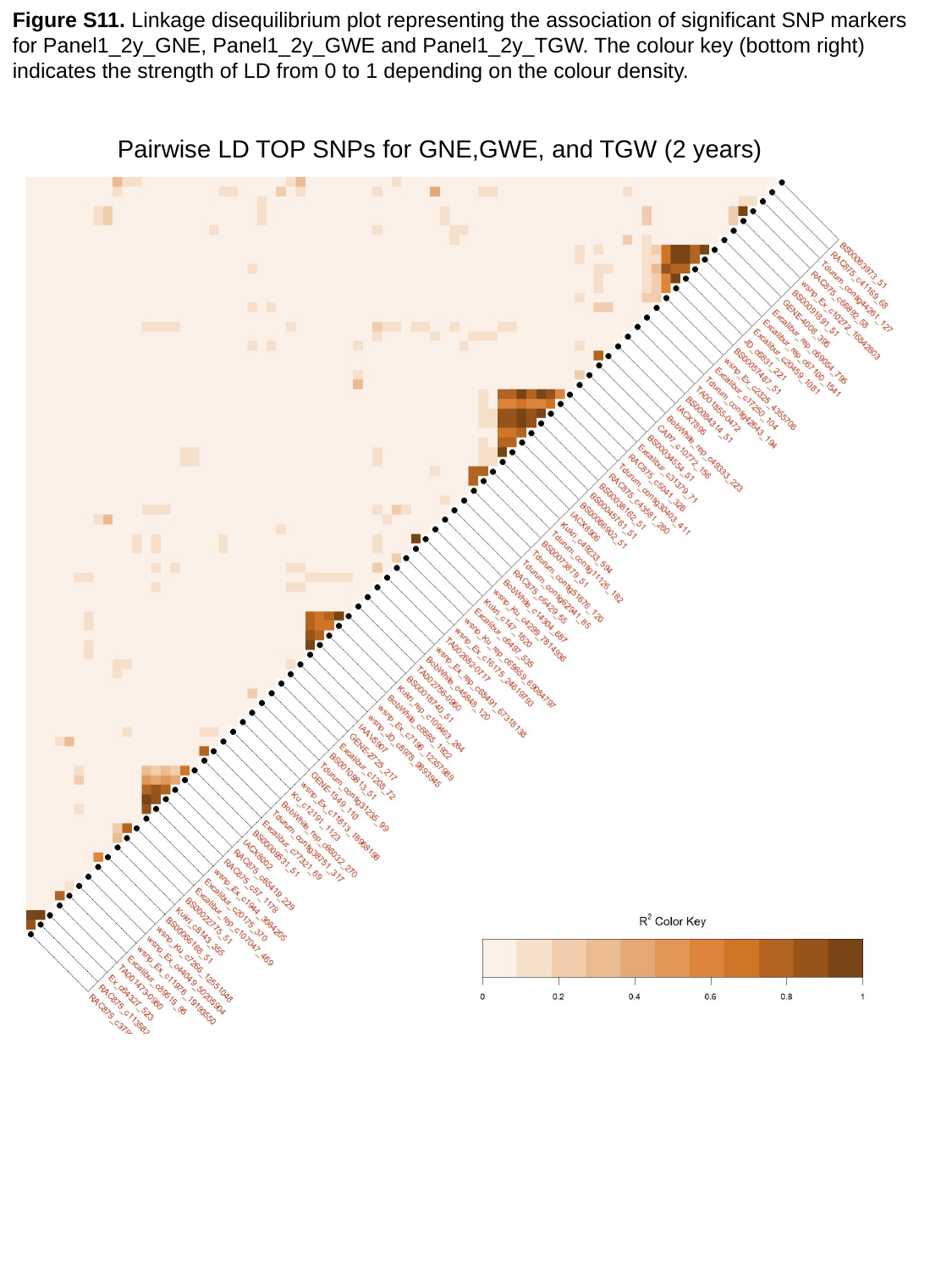

Figure S11. Linkage disequilibrium plot representing the association of significant SNP markers for Panel1_2y_GNE, Panel1_2y_GWE and Panel1_2y_TGW. The colour key (bottom right) indicates the strength of LD from 0 to 1 depending on the colour density.
Pairwise LD TOP SNPs for GNE,GWE, and TGW (2 years)

## Slide 12
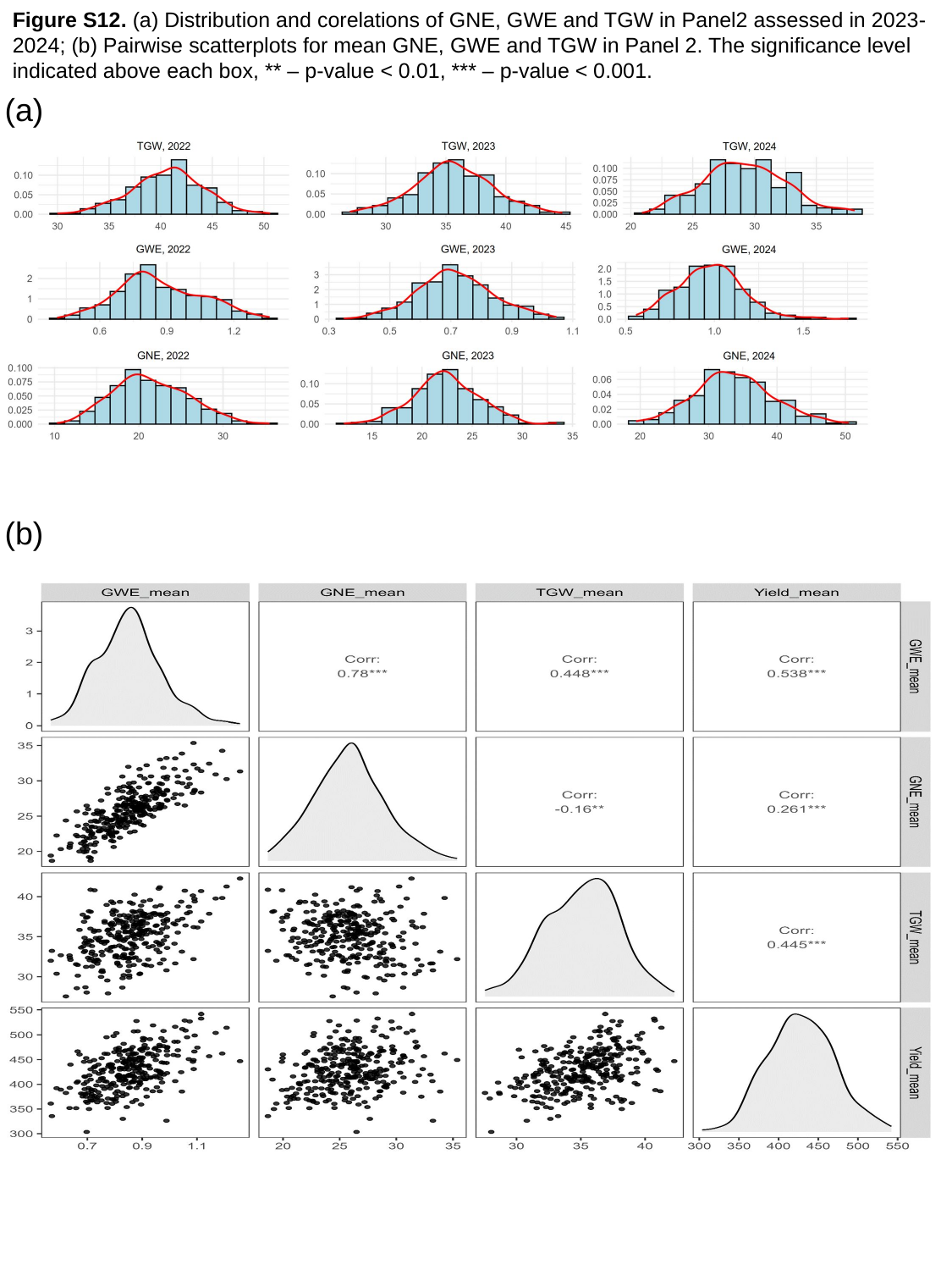

Figure S12. (a) Distribution and corelations of GNE, GWE and TGW in Panel2 assessed in 2023-2024; (b) Pairwise scatterplots for mean GNE, GWE and TGW in Panel 2. The significance level indicated above each box, ** – p-value < 0.01, *** – p-value < 0.001.
(a)
(b)
